# Soil prokaryotic and fungal biome structures associated with crop disease status across the Japan Archipelago

**DOI:** 10.1128/msphere.00803-23

**Published:** 2024-04-03

**Authors:** Hiroaki Fujita, Shigenobu Yoshida, Kenta Suzuki, Hirokazu Toju

**Affiliations:** 1 Center for Ecological Research, Kyoto University, Otsu, Shiga, Japan; 2 Institute for Plant Protection, National Agriculture and Food Research Organization, Tsukuba, Ibaraki, Japan; 3 Integrated Bioresource Information Division, BioResource Research Center, Tsukuba, Ibaraki, Japan; 4 Center for Living Systems Information Science (CeLiSIS), Graduate School of Biostudies, Kyoto University, Kyoto, Japan; 5 Laboratory of Ecosystems and Coevolution, Graduate School of Biostudies, Kyoto University, Kyoto, Japan; University of South Africa, Johannesburg, Gauteng, South Africa

**Keywords:** alternative stable states, community stability, ecosystem functions, keystone species, meta-analysis, microbial interactions, network hubs, plant pathogens

## Abstract

Archaea, bacteria, and fungi in the soil are increasingly recognized as determinants of agricultural productivity and sustainability. A crucial step for exploring soil microbiomes with important ecosystem functions is to perform statistical analyses on the potential relationship between microbiome structure and functions based on comparisons of hundreds or thousands of environmental samples collected across broad geographic ranges. In this study, we integrated agricultural field metadata with microbial community analyses by targeting 2,903 bulk soil samples collected along a latitudinal gradient from cool-temperate to subtropical regions in Japan (26.1–42.8 °N). The data involving 632 archaeal, 26,868 bacterial, and 4,889 fungal operational taxonomic units detected across the fields of 19 crop plant species allowed us to conduct statistical analyses (permutational analyses of variance, generalized linear mixed models, randomization analyses, and network analyses) on the relationship among edaphic factors, microbiome compositions, and crop disease prevalence. We then examined whether the diverse microbes form species sets varying in potential ecological impacts on crop plants. A network analysis suggested that the observed prokaryotes and fungi were classified into several species sets (network modules), which differed substantially in association with crop disease prevalence. Within the network of microbe-to-microbe coexistence, ecologically diverse microbes, such as an ammonium-oxidizing archaeon, an antibiotics-producing bacterium, and a potentially mycoparasitic fungus, were inferred to play key roles in shifts between crop-disease-promotive and crop-disease-suppressive states of soil microbiomes. The bird’s-eye view of soil microbiome structure will provide a basis for designing and managing agroecosystems with high disease-suppressive functions.

**IMPORTANCE:**

Understanding how microbiome structure and functions are organized in soil ecosystems is one of the major challenges in both basic ecology and applied microbiology. Given the ongoing worldwide degradation of agroecosystems, building frameworks for exploring structural diversity and functional profiles of soil microbiomes is an essential task. Our study provides an overview of cropland microbiome states in light of potential crop-disease-suppressive functions. The large data set allowed us to explore highly functional species sets that may be stably managed in agroecosystems. Furthermore, an analysis of network architecture highlighted species that are potentially used to cause shifts from disease-prevalent states of agroecosystems to disease-suppressive states. By extending the approach of comparative analyses toward broader geographic ranges and diverse agricultural practices, agroecosystem with maximized biological functions will be further explored.

## INTRODUCTION

The ongoing global-scale degradation of agroecosystems is threatening food production (1, 2). Maximizing the functions of microbial communities (microbiomes) is a prerequisite for building bases of sustainable agriculture (3–7). Archaea, bacteria, and fungi in the soil drive cycles of carbon, nitrogen, and phosphorus within agroecosystems (8–12). Many of those microbes also work to promote crop plant’s tolerance to drought and high temperature stresses as well as resistance to pests and pathogens (13–18). Importantly, those microbes vary greatly in their physiological impacts on crop plants (19–21). Therefore, gaining insights into soil microbiome compositions and functions is an essential starting point for managing resource-use efficient and disease-tolerant agroecosystems.

Since the emergence of high-throughput DNA sequencing, a number of studies have revealed taxonomic compositions of prokaryotes and/or fungi in agroecosystem soil (22–24). Those studies have explored microbial species that potentially support crop plant growth and/or prevent crop plant disease (9, 16, 25, 26). Meanwhile, each of the previous studies has tended to focus on specific crop plant species in specific farm fields (27), although there are some exceptionally comprehensive studies comparing multiple research sites (15, 22, 28). Therefore, generality in relationship between microbiome structure and functions remains to be examined in broader contexts (cf. global-scale analyses of soil microbiomes in natural ecosystems [29–32]). In other words, we still have limited knowledge of general patterns and features common to soil microbiomes with high crop plant yield or those with least crop disease risk. Thus, statistical analyses comparing microbiome structure among diverse crop plants across broad geographic ranges (15, 22) are expected to deepen our understanding of microbial functions in agroecosystems. In particular, comparative studies of thousands of soil samples covering a wide range of latitudes will provide opportunities for finding general properties common to microbial communities with plant-growth-promoting or crop-disease-suppressive functions across diverse climatic conditions.

Large data sets of soil microbiomes will also allow us to estimate interspecific interactions between microbial species (3, 33, 34). Archaea, bacteria, and fungi in soil ecosystems potentially form entangled webs of facilitative or competitive interactions, collectively determining ecosystem-level functions such as the efficiency of nutrient cycles and the prevalence of plant pathogens (35, 36). In fact, ecological network studies have inferred how sets of microbial species could respond to the outbreaks or experimental introductions of crop plant pathogens (37–39). Although various statistical platforms for deciphering the architecture of such microbial interaction networks have been proposed (33, 40), hundreds or more of microbial community samples are required to gain reliable inferences on interactions that reproducibly occur in real ecosystems (41). Thus, data sets consisting of thousands of soil samples collected across a number of local ecosystems will provide fundamental insights into how soil ecological processes are driven by cross-kingdom interactions involving archaea, bacteria, and fungi.

In this study, we conducted a comparative analysis of agroecosystem soil microbiomes based on 2,903 bulk soil samples collected from subtropical to cool-temperate regions across the Japan Archipelago. Based on the amplicon sequencing data set representing farm fields of 19 crop plant species, we profiled prokaryotic and fungal community compositions in conventional agricultural fields in Japan. By compiling the metadata of the soil samples, we examined the potential relationship between soil microbiome structure and the prevalence of crop disease. The microbiome data set was then used to infer the structure of a microbe-to-microbe coexistence network consisting of diverse archaea, bacteria, and fungi. Specifically, we examined whether the network architecture was partitioned into compartments (modules) of closely interacting microbial species. In addition, we tested the hypothesis that such network modules could differ in their positive/negative associations with crop plant disease/health status. To explore prokaryotic and fungal species keys to manage agroecosystem structure and functions, we further explored “core” or “hub” species that were placed at the central positions within the inferred microbial interaction network. Overall, this study provides an overview of soil microbial diversity of cropland soil across a latitudinal gradient, setting a basis for diagnosing soil ecosystem status and identifying sets of microbes to be controlled in sustainable crop production.

## RESULTS

### Diversity of agroecosystem microbiomes

**Fig 1:**
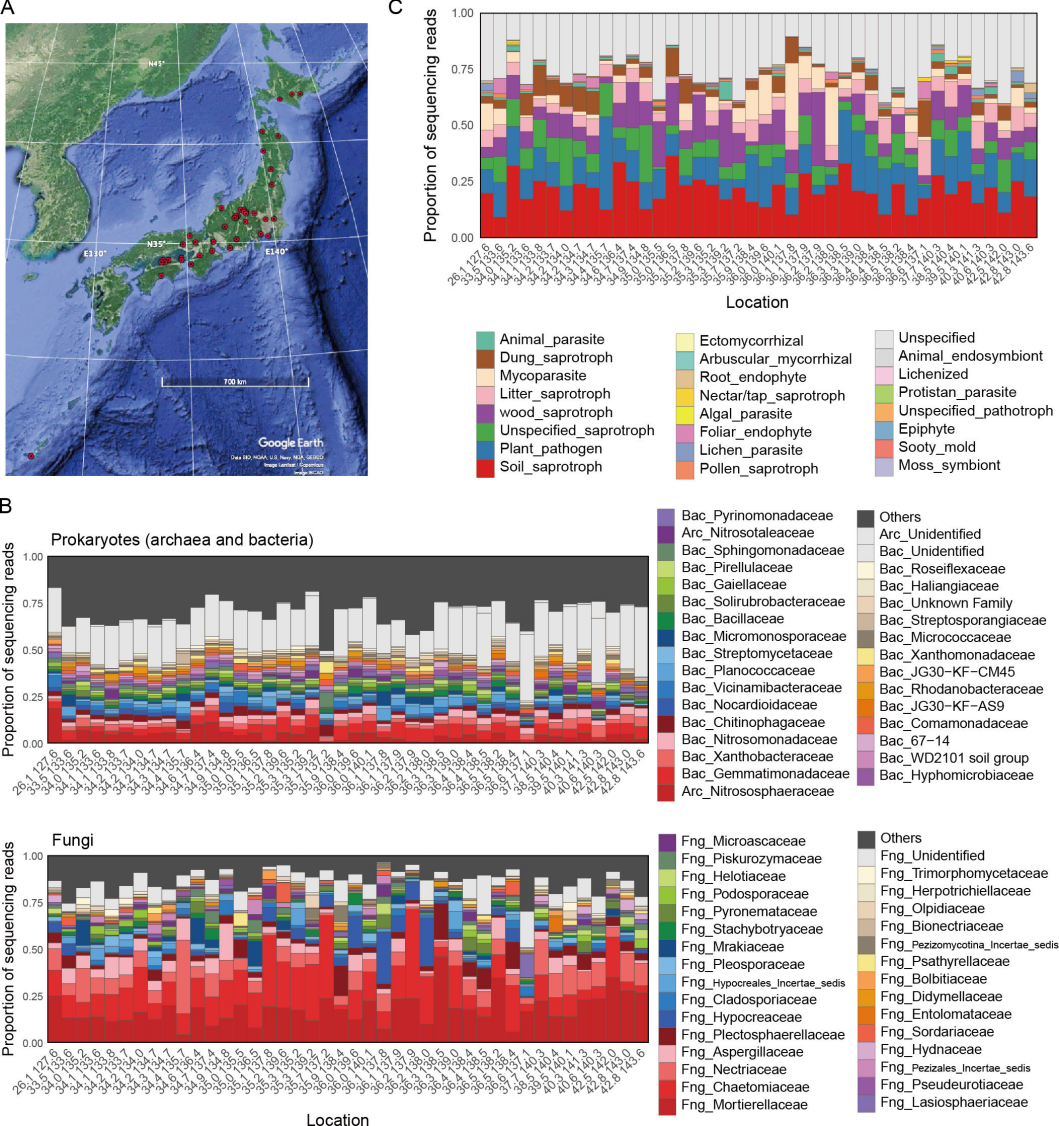
Comparison of soil microbiome structure across the Japan Archipelago. (**A**) Map of research sites across the Japan Archipelago. The 2,903 soil samples were grouped into 42 research sites when their latitude and longitude profiles were rounded to one decimal place. (**B**) Taxonomic compositions of prokaryotes (archaea and bacteria; top) and fungi (bottom) at the family level. See Fig. S1 for results at the genus, order, and class levels. (**C**) Compositions of functional groups of fungi.

We compiled the field metadata of 2,903 bulk soil samples collected in the research projects of National Agricultural and Food Research Organization (NARO), Japan. The bulk soil of farmlands was sampled from subtropical to cool-temperate regions (26.1–42.8 °N) across the Japan Archipelago from 2006 to 2014, targeting 19 crop plant species (Fig. 1A; Data S1). Most of the croplands were managed with conventional agricultural practices (characterized by intensive tillage and chemical fertilizer/pesticide application), while some were experimentally controlled as organic agricultural fields. The metadata (Data S1) included the information of chemical [e.g., pH, electrical conductivity, carbon/nitrogen (C/N) ratio, and available phosphorous concentration], physical (e.g., soil taxonomy), and biological (e.g., crop disease level) properties, providing a platform for profiling ecosystem states of cropland soil.

To integrate the metadata with the information of microbial community structure, we performed DNA metabarcoding analyses of both prokaryotes (archaea and bacteria) and fungi. After a series of quality filtering, prokaryotic and fungal community data were obtained from 2,676 and 2,477 samples, respectively. In total, 632 archaeal operational taxonomic units (OTUs) representing 22 genera (24 families), 26,868 bacterial OTUs representing 1,120 genera (447 families), and 4,889 fungal OTUs representing 1,190 genera (495 families) were detected (Fig. 1B; Fig. S1).

The prokaryotic communities lacked apparently dominant taxa at the genus and family levels (Fig. 1B). In contrast, the fungal communities were dominated by fungi in the families Mortierellaceae, Chetomiaceae, and Nectriaceae, depending on localities (Fig. 1B). A reference database profiling of fungal functional groups suggested that the fungal communities were dominated by soil saprotrophic and plant pathogenic fungi (Fig. 1C) as characterized by the dominance of *Mortierella* and *Fusarium* at the genus level (Fig. S1). Meanwhile, mycoparasitic fungi had exceptionally high proportions at some research sites, as represented by the dominance of *Trichoderma* (Hypocreaceae) at those sites (Fig. 1B; Fig. S1).

### Microbiome structure and crop disease prevalence

**Fig 2:**
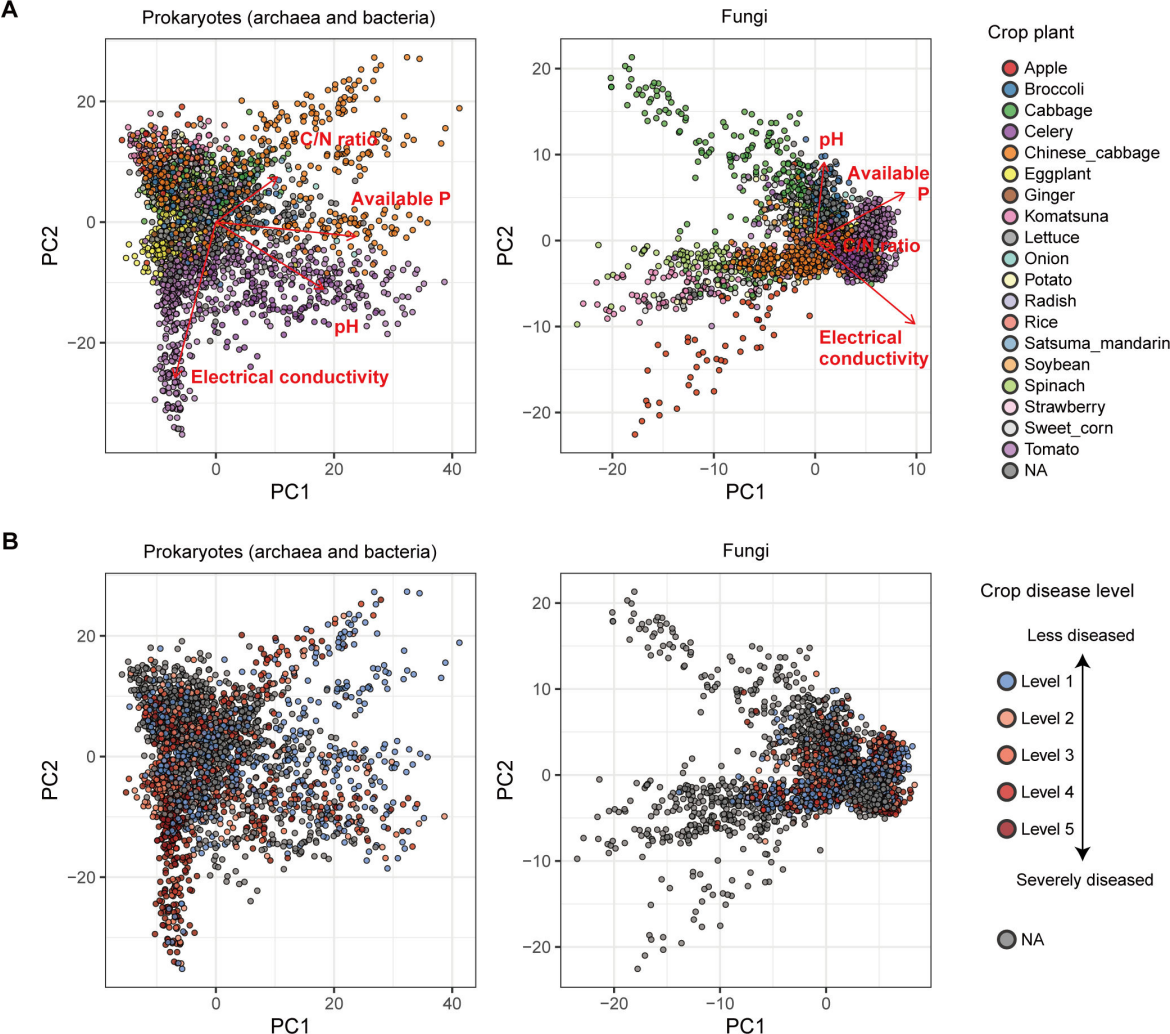
Dimensions of soil microbiome structure. (**A**) Prokaryote and fungal community structure. Principal co-ordinate analyses (PCA) were performed based on OTU-level compositional matrices, respectively, for the prokaryotic and fungal communities. The identify of crop plants is shown by colors. See Fig. S2 and S3 for relationship between community structure and environmental factors. Vectors with maximum correlations with respective soil environmental variables are shown as arrows. (**B**) Crop disease level and microbial community structure. On the PCA surface, crop disease level (see Materials and Methods) is indicated.

Compiling the metadata of edaphic factors, we found that variation in the community structure of prokaryotes and fungi was significantly explained by crop plant identity and soil taxonomy as well as by soil chemical properties such as pH, electrical conductivity, and C/N ratio (Fig. 2A; Fig. S2 and S3; Table 1). In addition, the ratio of prokaryotic abundance to fungal abundance, which was estimated based on a quantitative amplicon sequencing technique (42) (see Materials and Methods for details), was associated with both prokaryotic and fungal community structure (Table 1). The prokaryotic and fungal community structure also showed variation along latitude (Fig. S4; Table 1). Nonetheless, the explanatory powers of these variables were all small as indicated by the low *R*^2^ values (Table 1).

**TABLE 1:** Effects of environmental variables on prokaryotic/fungal community structure^*a*^

Model	Data set	Variable	df	*R* ^2^	*F*	*P*
Categorical variables	Prokaryotes	Research site	34	0.051	5.13	<0.001
		Month	11	0.014	4.36	<0.001
		Crop	16	0.006	1.21	0.030
		Former crop	22	0.024	3.78	<0.001
		Soil category	10	0.006	2.12	<0.001
		Residual	2,301	0.673		
		Total	2,400	1.000		
	Fungi	Research site	33	0.064	6.91	<0.001
		Month	11	0.012	3.96	<0.001
		Crop	15	0.006	1.54	<0.001
		Former crop	20	0.023	4.14	<0.001
		Soil category	10	0.005	1.75	<0.001
		Residual	2,109	0.591		
		Total	2,206	1.000		
Continuous variables	Prokaryotes	pH	1	0.011	16.52	<0.001
		Electrical conductivity	1	0.009	14.09	<0.001
		Available *P*	1	0.009	13.81	0.626
		C/N ratio	1	0.004	6.19	<0.001
		Latitude	1	0.006	9.00	<0.001
		Longitude	1	0.008	11.41	<0.001
		Prokaryote/fungus ratio	1	0.004	5.98	<0.001
		Residual	1,408	0.936		
		Total	1,415	1.000		
	Fungi					
		pH	1	0.013	19.64	<0.001
		Electrical conductivity	1	0.011	17.49	<0.001
		Available *P*	1	0.009	13.38	0.477
		C/N ratio	1	0.008	12.74	<0.001
		Latitude	1	0.016	25.58	<0.001
		Longitude	1	0.017	26.37	0.230
		Prokaryote/fungus ratio	1	0.009	13.61	<0.001
		Residual	1,408	0.904		
		Total	1,415	1.000		

^*a*^ For each set of categorical/continuous environmental variables, a PERMANOVA was performed for each of the prokaryotic and fungal community data sets.

Both prokaryotic and fungal community structures were significantly associated with the severity of crop disease (Fig. 2B; Table 2). Specifically, the crop plants’ disease/health status (disease level 1 vs disease levels 2–5; see Materials and Methods) was explained by some of the principal components (PCs) defined based on prokaryotic/fungal community structure (Fig. 2).

**TABLE 2:** Relationship between prokaryotic/fungal community structure on the disease level of crop plants^*a*^

Data set	Variable	*z*	*P*
Prokaryotes (*N* = 1,379)	PC1	1.59	0.1111
	PC2	−1.65	0.1000
	PC3	1.82	0.0684
	PC4	−2.32	0.0205
	PC5	3.98	0.0001
Fungi (*N* = 1,320)	PC1	1.52	0.1281
	PC2	1.39	0.1656
	PC3	−2.11	0.0348
	PC4	2.62	0.0089
	PC5	−0.84	0.4002

^*a*^ A generalized linear mixed model (GLMM) of crop plants’ disease level (disease level 1 vs disease levels 2–5) with a logit-link function and binomial errors was constructed by setting principal components of prokaryotic/fungal community structure (Fig. 2) as explanatory variables (fixed effects). The identity of experimental/research purposes, sampling month, and crop plant species was included as random effects in the GLMM.

### Microbes associated with crop disease/health status

We explored microbial OTUs whose prevalence is associated with crop plant disease/health status. Based on a randomization analysis, prokaryotic/fungal OTUs whose distribution is biased in samples representing the minimal crop disease level (disease level 1) were screened (Fig. S5).

**Fig 3:**
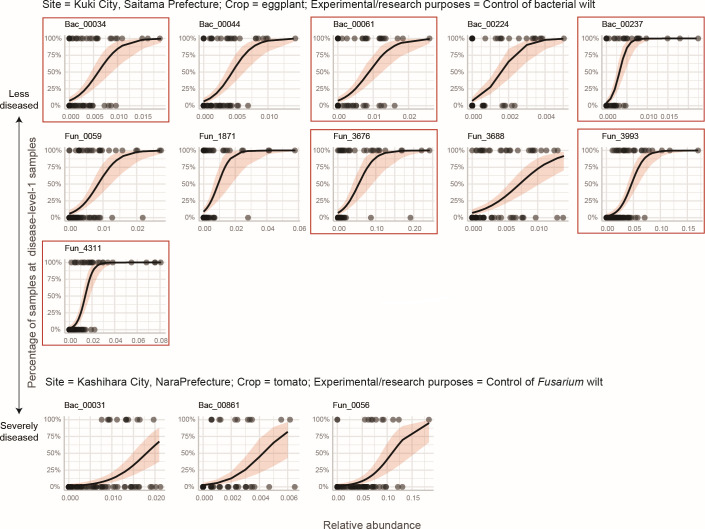
Relationship between OTU abundance and crop plant health. Among the six sub-data sets representing unique crop plant × site combinations, OTUs showing strongest specificity to the minimal crop disease level (*z*-standardized specificity to disease level 1 ≥ 6.0) were observed in two sub-data sets (“eggplant in Kuki City” and “tomato in Kashihara City”; Table 4; see Data S2 for full results). For each OTU in each sub-data set, generalized linear model with a logit function and binomial errors was constructed to examine relationship between OTU relative abundance and crop disease level (level 1 vs levels 2–5). All the regression lines are statistically significant (FDR <0.0001). The OTUs exhibiting statistically significant specificity to disease level 1 in the analysis with the entire data set (FDR <0.025; two-tailed test; Fig. S5 and S6) are highlighted with red squares.

To examine whether the OTUs highlighted in the across-Japan spatial scale could actually show tight associations with crop disease status at local scales, the randomization analysis was performed as well in each of the six sub-data sets representing unique combinations of research sites, crop plant species, and experimental/research purposes (Data S2). Statistically significant specificity for crop disease level [false discovery rate (FDR) <0.025; two-tailed test] was observed for at least one OTU in five of the six sub-data sets (Data S2). Among them, exceptionally strong specificity to the lowest crop disease level (standardized specificity score ≥6.0; FDR <0.0001) was detected in two sub-data sets (Table 3). The relative abundance of these OTUs was tightly associated with crop disease level across samples within each sub-data set (Fig. 3).

**TABLE 3:** Prokaryotic and fungal OTUs showing highest associations with crop health status within local croplands^*a*^

				Specificity to disease level 1		BLAST top-hit results				
Site	Crop	Experiment/research identity	ID	Score	FDR	Scientific name	Query cover (%)	*E* value	Identity (%)	Accession
Kuki	Eggplant	Control of bacterial wilt	Bac_00034	6.53	<0.0001	*Denitratisoma oestradiolicum*	100	9.0E−103	95.1	KF810120.1
			Bac_00044	6.67	<0.0001	*Nocardioides cynanchi*	100	4.0E−121	99.6	CP044344.1
			Bac_00061	7.12	<0.0001	*Piscinibacter aquaticus*	100	4.0E−121	99.6	KY284087.1
			Bac_00224	7.52	<0.0001	*Dongia* sp.	100	9.0E−123	100.0	AB835804.1
			Bac_00237	8.76	<0.0001	*Chondromyces robustus*	100	2.0E−89	91.8	AJ233942.2
			Fun_0059	6.83	<0.0001	*Moesziomyces aphidis*	100	9.0E−123	100.0	MH777069.1
			Fun_1871	6.27	<0.0001	*Pseudeurotium bakeri*	100	3.0E−122	100.0	MK911621.1
			Fun_3676	7.72	<0.0001	*Cladosporium proteacearum*	100	8.0E−118	100.0	OR857360.1
			Fun_3688	6.38	<0.0001	*Nigrospora sphaerica*	100	8.0E−118	100.0	OP113684.1
			Fun_3993	7.01	<0.0001	*Fusarium equiseti*	100	3.0E−112	99.6	MT588081.1
			Fun_4311	8.75	<0.0001	*Gibellulopsis nigrescens*	100	5.0E−110	100.0	OP498056.1
Kashihara	Tomato	Control of *Fusarium* wilt	Bac_00031	6.74	<0.0001	*Ramlibacter algicola*	100	9.0E−123	100.0	NR_175506.1
			Bac_00861	6.98	<0.0001	*Rhizomicrobium* sp.	100	4.0E−116	98.4	LN876448.1
			Fun_0056	6.28	<0.0001	*Corynascus sepedonium*	100	9.0E−123	100.0	OW986289.1

^*a*^ Among the six sub-data sets representing unique combinations of research sites, crop plant species, and research experimental/research purposes, OTUs showing strongest specificity to the minimal crop disease level (*z*-standardized specificity to disease level 1 ≥ 6.0) were observed in two sub-data sets (“eggplant in Kuki City” and “tomato in Kashihara City”). The OTUs are shown with the NCBI BLAST top-hit results. See Data S2 for the full results.

### Microbe-to-microbe network

**Fig 4:**
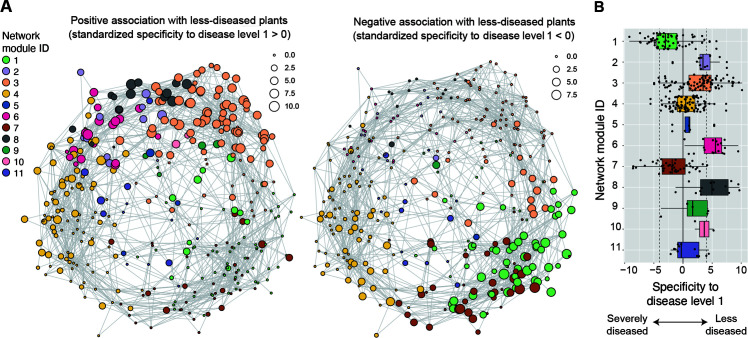
Architecture of microbe-to-microbe network. (**A**) Co-occurrence networks of archaea, bacteria, and fungi. Specificity of occurrences to disease-level-1 (the lowest disease level) samples (Fig. S5 and S6) is shown for each OTU within the network. The specificity is shown as node size separately for positive (left) and negative (right) associations with lowest-diseased states of crop plants. Colors indicate network modules, in which microbial OTUs in commensalistic/mutualistic interactions and/or those sharing environmental preferences are densely linked with each other. See Fig. S9 for taxonomy (archaea, bacteria, or fungi) of respective nodes. (**B**) Characteristics of network modules. Mean specificity to the minimal crop disease level (disease level 1; left in panel A) is shown for each network module.

We then examined the network architecture of potential microbe-to-microbe interactions within the soil microbiomes. The inferred network of coexistence was subdivided into several modules, in which archaeal, bacterial, and fungal OTUs sharing environmental preferences and/or those in positive interactions were linked with each other (Fig. 3A; Fig. S6 to S9). The network modules differed considerably in their association with crop-plant disease level (Fig. 4B; Fig. S6; Data S3). Modules 2, 6, and 8, for example, were characterized by microbes associated with the lowest disease level. Module 6, which showed the highest mean specificity to the minimal crop disease level (Fig. 4B), included a bacterial OTU allied to the genus *Gemmatimonas* (Bac_00025) that allied to the genus *Thermanaerothrix* (Bac_00258) and a fungal OTU assigned to the genus *Plectosphaerella* (Fun_4447) (Table 4). In contrast to these modules, modules 1 and 7 were constituted by microbes negatively associated with crop plant health (Fig. 4B). Module 1 included a bacterial OTU distantly allied to the genus *Ureibacillus* (Bac_00165) that assigned to the genus *Nonomuraea* (Bac_00004) and that assigned to the genus *Streptomyces* (Bac_00010), while module 7 involved a fungal OTU assigned to the genus *Fusarium* (Fun_4028) and an archaeal OTU assigned to the genus *Nitrososphaera* (Arc_006) (Table 4).

**TABLE 4:** Representative prokaryotic and fungal OTUs in network modules with highly positive/negative associations with crop plant health^*a*^

		Specificity to disease level 1		Network scores				BLAST top-hit results				
Module	OTU ID	Score	FDR	Degree	Betweenness	Within-module degree	Module connectivity	Scientific name	Query cover (%)	*E* value	Identity (%)	Accession
Module 2 (positive)	Bac_00147	7.526	<0.0001	4	0.001	−0.693	0.625	*Botrimarina hoheduenensis*	99	2.0E−90	92.2	NR_173585.1
	Bac_00469	6.307	<0.0001	5	0.001	−1.214	0.720	*Luteitalea pratensis*	100	9.0E−108	96.3	NR_156918.1
	Bac_00061	4.657	<0.0001	3	0.001	−1.214	0.667	*Piscinibacter aquaticus*	100	4.0E−121	99.6	KY284087.1
	Bac_00002	4.652	<0.0001	10	0.019	0.347	0.720	*Arthrobacter globiformis*	100	9.0E−123	100.0	OR780585.1
	Bac_00463	4.166	<0.0001	5	0.002	0.347	0.320	*Flavobacterium sufflavum*	100	9.0E−123	100.0	NR_171469.1
Module 6 (positive)	Bac_00025	9.477	<0.0001	5	0.001	0.189	0.000	*Gemmatimonas aurantiaca*	100	4.0E−86	91.0	KF228166.1
	Bac_00258	7.205	<0.0001	14	0.011	2.587	0.357	*Thermanaerothrix daxensis*	100	4.0E−76	88.6	NR_117865.1
	Fun_4447	7.151	<0.0001	10	0.017	−0.610	0.780	*Plectosphaerella cucumerina*	100	2.0E−108	100.0	MT529301.1
	Fun_0071	7.093	<0.0001	6	0.003	−1.009	0.722	*Schizothecium miniglutinans*	100	2.0E−104	95.5	MW472119.1
	Bac_00182	6.787	<0.0001	21	0.037	1.788	0.649	*Brevitalea aridisoli*	100	9.0E−93	92.7	NR_151987.1
Module 8 (positive)	Bac_00294	9.679	<0.0001	7	0.002	0.000	0.571	*Luteitalea pratensis*	100	4.0E−81	89.8	NR_156918.1
	Bac_00171	9.499	<0.0001	11	0.012	1.063	0.628	*Membranihabitans marinus*	100	9.0E−88	91.4	OL441066.1
	Fun_4070	8.282	<0.0001	10	0.006	−0.532	0.700	*Mortierella* sp.	80	3.0E−87	99.5	OP799297.1
	Bac_00138	7.904	<0.0001	20	0.023	1.595	0.715	*Ectothiorhodospira mobilis*	100	4.0E−86	91.0	MG264243.1
	Bac_00339	7.297	<0.0001	5	0.003	−1.063	0.480	*Vicinamibacter silvestris*	100	2.0E−74	88.2	NR_151905.1
Module 1 (negative)	Bac_00165	−9.211	<0.0001	19	0.035	0.903	0.582	*Ureibacillus suwonensis*	96	1.0E−76	89.5	JX914499.1
	Bac_00004	−7.370	<0.0001	17	0.021	1.134	0.512	*Nonomuraea phyllanthi*	100	9.0E−123	100.0	CP045572.1
	Bac_00010	−7.370	<0.0001	4	0.001	−0.714	0.375	*Streptomyces ardesiacus*	100	9.0E−123	100.0	OR873433.1
	Bac_00282	−7.289	<0.0001	9	0.009	0.672	0.000	*Hyphomicrobium aestuarii*	100	4.0E−111	97.1	NR_104954.1
	Bac_00036	−6.531	<0.0001	16	0.011	1.596	0.305	*Nitrolancea hollandica*	100	9.0E−88	91.5	MW367426.1
Module 7 (negative)	Fun_4028	−8.585	<0.0001	5	0.000	−0.490	0.320	*Fusarium oxysporum*	100	6.0E−114	100.0	MF281350.2
	Arc_006	−8.544	<0.0001	8	0.006	−0.490	0.594	*Nitrososphaera viennensis*	100	9.0E−123	100.0	NR_134097.1
	Bac_00195	−8.079	<0.0001	14	0.019	1.218	0.449	*Chromobacterium amazonense*	100	3.0E−88	91.5	OQ061977.1
	Bac_00062	−7.281	<0.0001	13	0.011	1.218	0.379	*Sphingomonas segetis*	100	4.0E−116	98.4	NR_175421.1
	Bac_00098	−6.557	<0.0001	18	0.026	1.218	0.537	*Geodermatophilus normandii*	100	9.0E−118	98.8	MT214187.1

^*a*^ In each of the modules 2, 6, and 8 (Fig. 4), the top 5 OTUs with the highest specificity to the minimal crop disease level (specificity to disease level 1; see Fig. S5 for the relationship between the specificity score and FDR). For each OTU, network degree, betweenness centrality, within-module degree (*z*-standardized), and among-module connectivity (Fig. 6) are presented with the NCBI BLAST top-hit results. Likewise, in each of the modules 1 and 7 (Fig. 4), the top 5 OTUs negatively associated with the minimal crop disease level are shown. See Data S3 for the full results.

### Core species within the microbial network

**Fig 5:**
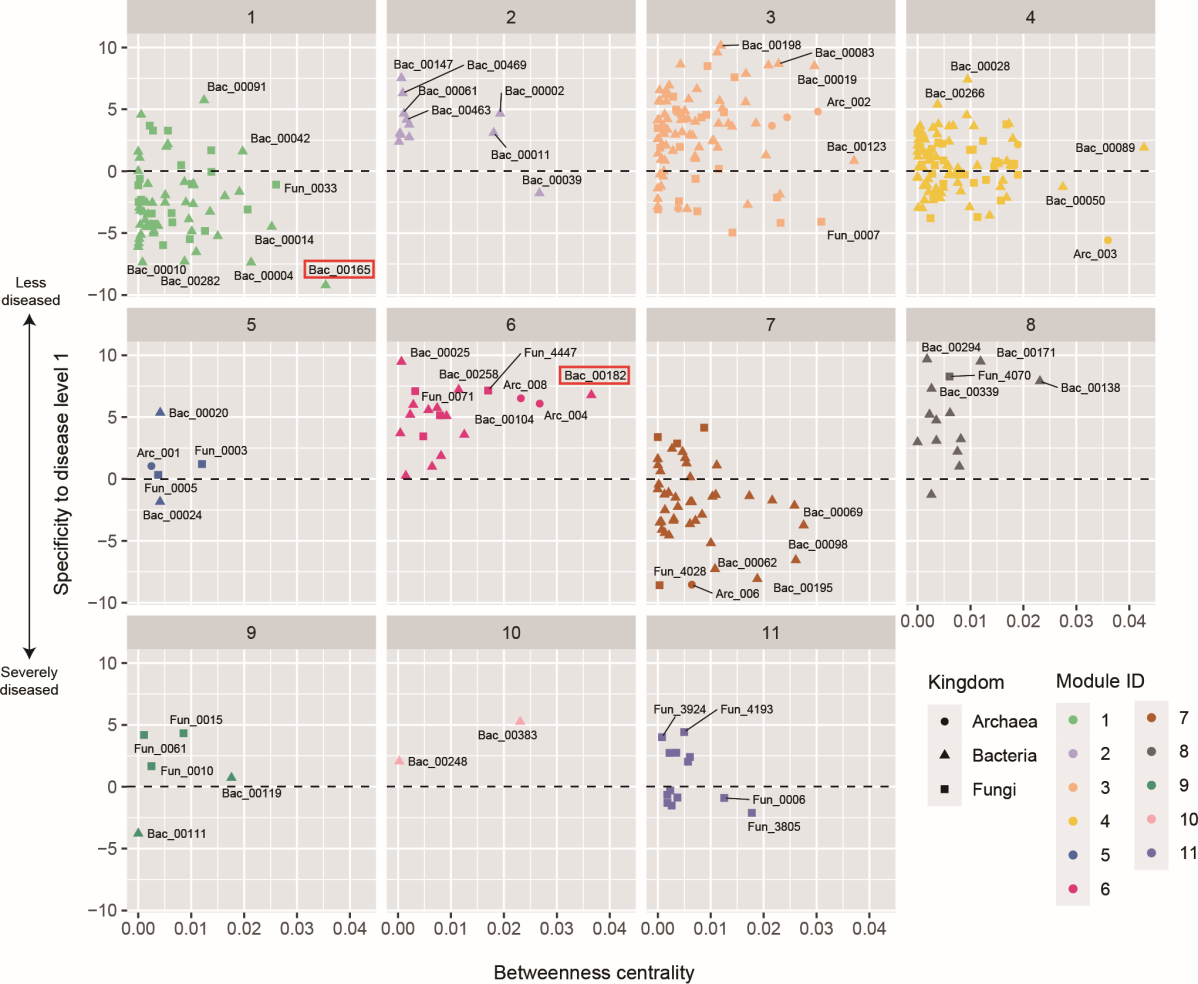
Properties of the microbe-to-microbe network modules. For each network module, specificity to the minimal crop disease level (disease level 1) is shown for each prokaryote/fungal OTU along the vertical axis. Betweenness centrality, which measures the extent to which an OTU is located within the shortest paths connecting pairs of other nodes in a network, is shown along the horizontal axis. The OTUs mentioned in the main text are highlighted with red squares.

We next explored microbial OTUs that potentially have great impacts on community- or ecosystem-scale processes based on an analysis of the microbe-to-microbe network architecture (Data S3). Among the microbes disproportionately found from the samples with the minimal crop disease level, a Pyrinomonadaceae bacterial OTU allied to the genus *Brevitalea* (Bac_00182 in module 6; Table 4), for example, showed a high betweenness centrality score (Fig. 5). Meanwhile, among the microbes negatively associated with crop health status, a bacterial OTU distantly allied to the genus *Ureibacillus* (Bac_00165 in module 1; Table 4) was inferred to be located at a central position within the network (Fig. 5).

**Fig 6:**
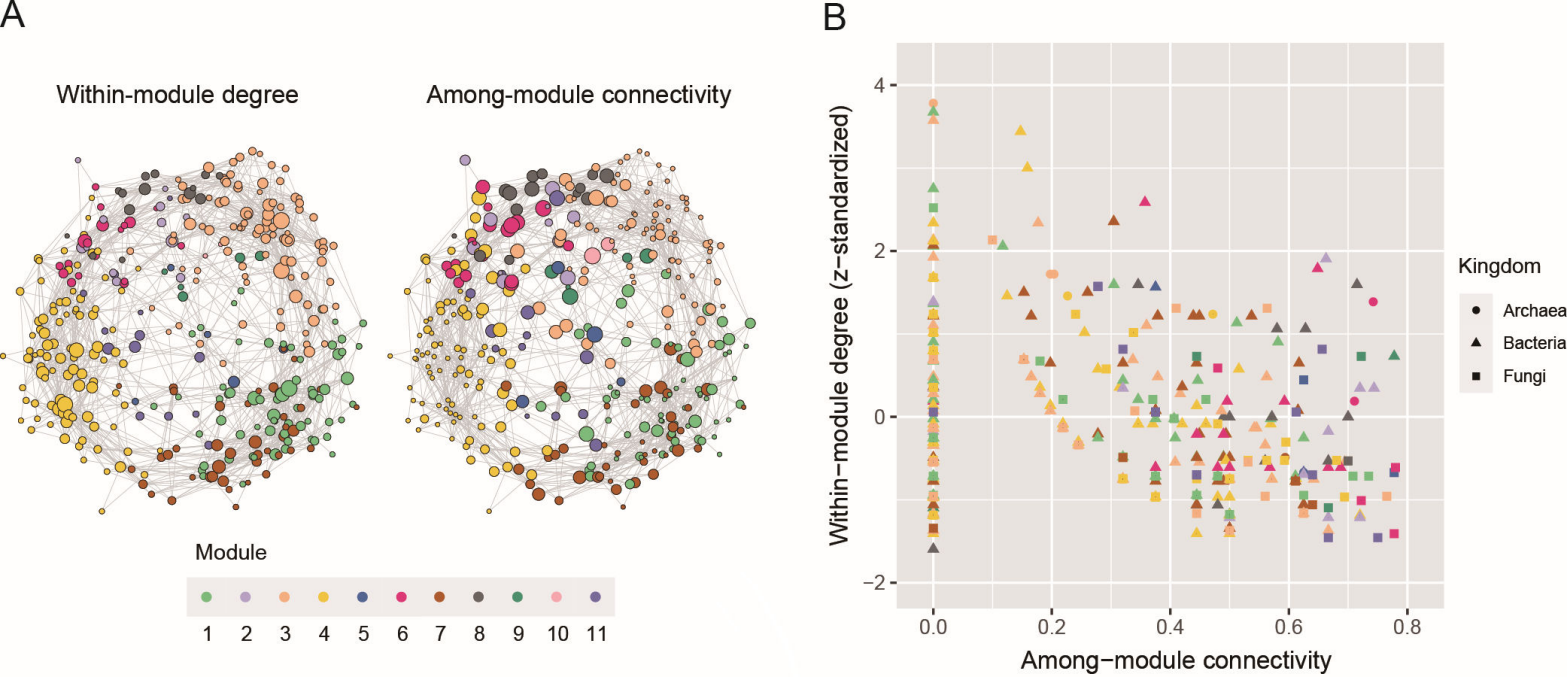
Topological roles of OTUs within and across network modules. (**A**) Position of potential hubs within the network. In each graph, node size roughly represents within-module degree (left) or among-module connectivity (right). (**B**) Network hub indices. For each OTU, within-module degree represents the number of OTUs linked with the target OTU within a module (*z*-standardized). Among-module connectivity represents the extent to which an OTU interlinks OTUs belonging to different network modules. The prokaryotic/fungal OTU with the highest within-module degree or among-module connectivity in each of the modules 1, 2, 6, 7, and 8 (highlighted in the main text and Table 4) is indicated with its OTU ID. See Table 5 for the taxonomic profiles of the OTUs.

We further ranked microbial OTUs in terms of their topological roles in interlinking multiple network modules. We then found that OTUs linked with many other OTUs within modules were not necessarily placed at the topological positions interconnecting different modules (Fig. 6). In module 6, which showed high specificity to the minimal crop disease level (Fig. 4), a bacterial OTU distantly allied to the genus *Thermanaerothrix* (Bac_00258) was designated as a “within-module hub,” while a fungal OTU assigned to the genus *Plectosphaerella* (Fun_4447) showed a high “among-module connectivity” score (Table 4). Likewise, in module 1, which consisted of many OTUs with negative associations with crop plant health (Fig. 4), a bacterial OTU allied to the genus *Gemmatimonas* (Bac_00258) had the highest numbers of within-module links, while a fungal OTU assigned to the genus *Curvularia* (Fun_0043) was inferred to be an among-module hub (Table 5). The list of microbial OTUs placed at the interface of modules (OTUs with high among-module connectivity scores) involved an archaeal OTU assigned to the genus *Nitrosotenuis*, bacterial OTUs assigned to the genera *Arenimonas*, *Arthrobacter*, and *Streptomyces* bacteria, and fungal OTUs assigned to the genera *Mortierella*, *Curvularia*, and *Trichoderma* (Table 5).

**TABLE 5:** Within- and among-module hubs in the network^*a*^

Focused			Specificity to disease level 1		Network scores				BLAST top-hit results				
Index	Module	OTU ID	Score	FDR	Degree	Betweenness	Within-module degree	Module connectivity	Scientific name	Query cover (%)	*E* value	Identity (%)	Accession
Within	Module 2 (positive)	Bac_00011	3.104	0.0013	13	0.018	1.907	0.663	*Pseudomonas izuensis*	100	9.0E−123	100.0	OR841525.1
		Bac_00057	2.741	0.0063	6	0.002	1.387	0.000	*Massilia violaceinigra*	100	9.0E−123	100.0	MT373681.1
		Bac_00002	4.652	<0.0001	10	0.019	0.347	0.720	*Arthrobacter globiformis*	100	9.0E−123	100.0	OR780585.1
	Module 6 (positive)	Bac_00258	7.205	<0.0001	14	0.011	2.587	0.357	*Thermanaerothrix daxensis*	100	4.0E−76	88.6	NR_117865.1
		Bac_00182	6.787	<0.0001	21	0.037	1.788	0.649	*Brevitalea aridisoli*	100	9.0E−93	92.7	NR_151987.1
		Arc_004	6.089	<0.0001	19	0.027	1.388	0.742	*Nitrosotenuis chungbukensis*	100	9.0E−123	100.0	CP130341.1
	Module 8 (positive)	Bac_00138	7.904	<0.0001	20	0.023	1.595	0.715	*Ectothiorhodospira mobilis*	100	4.0E−86	91.0	MG264243.1
		Bac_00260	5.320	<0.0001	9	0.006	1.595	0.346	*Crenobacter cavernae*	100	4.0E−96	93.5	CP031337.1
		Bac_00171	9.499	<0.0001	11	0.012	1.063	0.628	*Membranihabitans marinus*	100	9.0E−88	91.4	OL441066.1
	Module 1 (negative)	Bac_00014	−4.481	<0.0001	22	0.025	3.676	0.000	*Gemmatimonas phototrophica*	100	2.0E−89	91.8	CP011454.1
		Bac_00055	−5.245	<0.0001	18	0.015	2.752	0.000	*Microbispora rosea*	100	9.0E−123	100.0	MN826183.1
		Fun_0033	−1.088	0.1752	17	0.026	2.521	0.000	*Trichoderma atroviride*	100	9.0E−123	100.0	MN429074.1
	Module 7 (negative)	Bac_00045	−1.380	0.1103	17	0.017	2.356	0.304	*Pseudolabrys taiwanensis*	100	9.0E−113	97.6	CP031417.1
		Bac_00116	−3.636	0.0003	13	0.006	2.071	0.000	*Streptomyces spinosirectus*	100	9.0E−123	100.0	CP090447.1
		Bac_00218	−5.182	<0.0001	13	0.010	1.502	0.260	*Dyella ginsengisoli*	100	9.0E−123	100.0	KY228986.1
Among	Module 2 (positive)	Bac_00039	−1.782	0.0537	11	0.027	0.347	0.744	*Arenimonas daechungensis*	100	4.0E−116	98.4	NR_109442.1
		Bac_00002	4.652	<0.0001	10	0.019	0.347	0.720	*Arthrobacter globiformis*	100	9.0E−123	100.0	OR780585.1
		Bac_00469	6.307	<0.0001	5	0.001	−1.214	0.720	*Luteitalea pratensis*	100	9.0E−108	96.3	NR_156918.1
	Module 6 (positive)	Fun_4447	7.151	<0.0001	10	0.017	−0.610	0.780	*Plectosphaerella cucumerina*	100	2.0E−108	100.0	MT529301.1
		Fun_3979	5.139	<0.0001	6	0.008	−1.409	0.778	*Enterocarpus grenotii*	100	8.0E−113	99.6	OU989357.1
		Arc_004	6.089	<0.0001	19	0.027	1.388	0.742	*Nitrosotenuis chungbukensis*	100	9.0E−123	100.0	CP130341.1
	Module 8 (positive)	Bac_00138	7.904	<0.0001	20	0.023	1.595	0.715	*Ectothiorhodospira mobilis*	100	4.0E−86	91.0	MG264243.1
		Bac_00180	2.213	0.0224	10	0.008	0.000	0.700	*Rubrobacter spartanus*	100	4.0E−66	86.3	NR_158052.1
		Fun_4070	8.282	<0.0001	10	0.006	−0.532	0.700	*Mortierella kuhlmanii*	100	9.0E−53	84.5	MH860115.1
	Module 1 (negative)	Fun_0043	0.485	0.3420	7	0.008	−0.714	0.735	*Curvularia senegalensis*	100	9.0E−123	100.0	MT476857.1
		Fun_3610	1.694	0.0649	12	0.014	−0.714	0.708	*Acremonium alternatum*	100	2.0E−118	100.0	MT529342.1
		Bac_00042	1.601	0.0774	12	0.020	−0.252	0.625	*Fulvivirgaceae bacterium*	100	9.0E−108	96.3	OQ733332.1
	Module 7 (negative)	Fun_0027	2.861	0.0040	5	0.004	−1.059	0.640	*Trichoderma virens*	100	9.0E−123	100.0	MT530036.1
		Bac_00032	−1.253	0.1331	4	0.001	−1.059	0.625	*Streptomyces cyaneus*	100	9.0E−123	100.0	OR807486.1
		Bac_00070	2.191	0.0242	4	0.005	−1.059	0.625	*Luteimonas aestuarii*	100	9.0E−123	100.0	OQ255277.1

^*a*^ In each of the modules highlighted in Table 4 and Fig. 6, the top 3 OTUs with the highest within-module degree (*z*-standardized) or among-module connectivity are shown. See Data S3 for the full results.

## DISCUSSION

We here profiled the diversity of agroecosystem microbiome structure across a latitudinal gradient from cool-temperate to subtropical regions based on the analysis of >2,000 soil samples. As partially reported in previous studies comparing microbiome compositions across broad geographic ranges (15, 22), prokaryotic and fungal community structure varied depending on season, crop plant species, former crop identity, and background soil categories (Fig. 2A; Fig. S2; Table 1). In addition, soil chemical properties such as pH, electrical conductivity, and C/N ratio as well as the prokaryote/fungus abundance ratio significantly explained variation in microbiome structure (Fig. S3; Table 1). In contrast, available phosphorus concentrations had significant effects on neither prokaryotic nor fungal communities in the multivariate model (Table 1), suggesting that nitrogen cycles rather than phosphorous ones are more tightly linked with microbiome structure. The integration of the microbiome data sets with agricultural field metadata allowed us to perform statistical tests of potential relationships between microbiome structure and agroecosystem performance (Fig. 2; Table 2). A series of OTU-level analyses further highlighted taxonomically diverse prokaryotes and fungi showing strong positive or negative associations with crop health status (Fig. 3; Fig. S5; Table 3).

We then examined how these microbes differing in association with crop disease/health status form a network of coexistence. The architecture of the network involving diverse archaeal, bacterial, and fungal OTUs was highly structured, being partitioned into 11 modules (Fig. 4A). Intriguingly, the network modules varied considerably in constituent microbes’ association with crop disease levels (Fig. 4B). This result suggests that sets of microbes can be used to design soil microbiomes with crop-disease-suppressive functions. Among the detected modules, modules 2, 6, and 8 were of particular interest with regard to the assembly of microbial OTUs positively associated with crop health status (Fig. 4 and 5). In contrast, modules 1 and 7 were constituted mainly by microbial OTUs negatively associated with plant health (Fig. 4B). In particular, module 7 was characterized by the presence of a notorious plant pathogenic fungus, *Fusarium oxysporum* (43, 44; but see reference 45 for diversity of their impacts on plants). All these modules included both prokaryotes and fungi (Fig. S9; Data S3), illuminating the importance of inter-kingdom interactions (3, 34). The presence of microbial species sets differing in plant-associated ecological properties suggests that keeping specific sets of compatible prokaryotes and fungi is essential for maximizing the stability of agricultural production (3).

The analysis of network architecture further allowed us to explore core or hub species within the microbial network (Fig. 6). Because the microbes highlighted with the examined network indices occupy key positions interconnecting many other microbes (46), their increase/decrease is expected to have profound impacts on whole community processes (3, 33, 34). In particular, control or manipulation of microbes located at the central positions interlinking different network modules (41) (i.e., microbes with high among-module connectivity; Fig. 6B) may trigger drastic shifts in microbial community structure between disease-promotive and disease-suppressive states (3). The candidate list of such core species involved an ammonium-oxidizing archaeon (*Nitrosotenuis*) (47), an antibiotics-producing bacterium (*Streptomyces*) (48), a prevalent soil fungus (*Mortierella*) (49, 50), a potentially mycoparasitic fungus (*Trichoderma*) (51, 52), and fungi allied to plant pathogenic clades (*Curvularia* and *Plectosphaerella* [anamorph = *Fusarium*]) (53, 54) (Table 5). Given that many of the bacterial and fungal taxa listed above are culturable, experimental studies examining their ecological roles are awaited. Specifically, it would be intriguing to test whether substantial shifts in soil microbiome structure and functions can be caused by the introduction of those among-module hub microbes.

Although the data set across a latitudinal gradient provided an opportunity for gaining bird’s-eye insights into the structure and potential functions of soil microbiomes, the results should be interpreted carefully with the recognition of potential methodological shortcomings and pitfalls. First, the approach of geographic comparison *per se* does not give a firm basis for deciphering microbial community dynamics. To gain fundamental insights into microbiome dynamics, we need to perform time-series monitoring (42, 55, 56) of soil prokaryotic and fungal community compositions. Second, information of microbial communities alone does not provide comprehensive insights into agroecosystem soil states. Given that soil ecosystem processes are driven not only by microbes but also by nematodes, arthropods, earthworms, and protists (57–60), simultaneous analyses of all prokaryotic and eukaryotic taxa (61, 62) will help us infer whole webs of biological processes. Third, meta-analyses of agroecosystem performance across diverse crop fields require utmost care because there is no firm criterion commonly applicable to different crop plant species or different pest/pathogen species. As implemented in this study, effects of such difference may be partially controlled by including them as random variables in generalized linear mixed models (GLMMs; Table 2). Nonetheless, local-scale analyses targeting specific crop plant species and disease symptoms (Fig. 3; Table 3; Data S2) are necessary to gain reliable inferences of potential microbial functions. Fourth, along with the potential pitfall discussed above, network modules can differ not only in properties related to crop disease/health status but also in those associated with crop plant identity or cropland management (Fig. S6 to S7). Again, findings in broad-geographic-scale analyses need to be supplemented by insights from local-scale observations (Fig. 3). Fifth, amplicon sequencing approaches provide only indirect inference of biological functions. With the current capacity of sequencing and bioinformatic technologies, it is hard to assemble tens of thousands of microbial genomes based on the analysis of thousands of environmental samples. Furthermore, due to the paucity of the information of fungal ecology and physiology, it remains difficult to annotate high proportions of genes within fungal genomic data. Nonetheless, with the accumulation of methodological breakthroughs, shotgun sequencing of soil microbiomes will deepen our understanding of agroecosystem processes (63–65). Sixth, the cooccurrence network approach employed in this study did not allow us to separate direct and indirect interactions between microbes. Shotgun metagenomic sequencing analyses will provide detailed insights into the structure of metabolic interdependence among microbial species (66, 67). Seventh, in this study, full sets of metadata were not available for all the sequenced samples, inevitably decreasing the number of samples examined in some statistical modeling. Although substantial efforts had been made to profile cropland soils in the national projects in which the soil samples were collected, continuous efforts are required to gain further comprehensive insights into agroecosystem structure and functions.

Expanding the comparative microbiome analysis to different geographic regions and agroecosystem management practices will contribute to a more comprehensive understanding of microbiome structure and function. For example, comparison with soil agroecosystems in lower-latitudinal or higher-latitudinal regions or meta-analyses covering multiple continents will provide further comprehensive knowledge of the diversity of microbiome structure. In addition to extensions toward broader geographic ranges, those toward diverse agroecosystem management are of particular importance. Given that our samples were collected mainly from croplands managed with conventional agricultural practices, involvement of soil samples from regenerative or conservation agricultural fields (68–71) will reorganize our understanding of the relationship between microbiome compositions and functions. In conclusion, this data-driven research lays the groundwork for understanding fundamental mechanisms in soil ecosystems, offering innovative strategies for the design of sustainable agriculture.

## MATERIALS AND METHODS

### Soil samples and metadata

Over research projects of NARO, which were carried out through five national research programs funded by Ministry of Agriculture, Forestry and Fisheries, 2,903 rhizosphere/bulk soil samples were collected from conventional agricultural fields across the Japan Archipelago from 23 January 2006, to 28 July 2014 (Data S1). When the latitude and longitude of the sampling positions were rounded to one decimal place, 42 research sites were distinguished. Across the metadata of the 2,903 samples, the information of 19 crop plants, 34 former crop plants (including “no crop”), 13 soil taxonomic groups (e.g., “Andosol”), 60 experimental/research purposes (e.g., “soil comparison between organic and conventional management”) was described. Likewise, the metadata included the information of dry soil pH, electrical conductivity, carbon/nitrogen ratio, and available phosphorous concentration from 2,830, 2,610, 2,346, and 2,249 samples, respectively. In addition, the information of the severity of crop plant disease was available for 1,472 samples (tomato, 637 samples; Chinese cabbage, 336 samples; eggplant, 202 samples; celery, 97 samples; Broccoli, 96 samples, etc.). The values of the proportion of diseased plants or disease severity index (72) were normalized within the ranges from 0 to 100, and they were then categorized into five levels (level 1, 0–20; level 2, 20–40; level 3; 40–60; level 4, 60–80; level 5, 80–100). The plant pathogens examined in the disease-level evaluation were *Colletotrichum gloeosporioides* on the strawberry, *Fusarium oxysporum* on the celery, the lettuce, the strawberry, and the tomato, *Phytophthora sojae* on the soybean, *Plasmodiophora brassicae* on Cruciferae plants, *Pyrenochaeta lycopersici* on the tomato, *Pythium myriotylum* on the ginger, *Ralstonia solanacearum* on the eggplant and the tomato, and *Verticillium* spp. on Chinese cabbage. For continuous variables within the metadata, emergent outliers (mean + 5 SD) were converted into “NA” in the data matrix used in the following statistical analyses as potential measurement/recording errors. Unrealistic electrical conductivity records (>20) were converted into “NA” as well.

At each sampling position, five soil sub-samples collected from the upper layer (0–10 cm in depth) at five points (ca. 100 g each) were mixed. The mixed soil sample (ca. 500 g) was then sieved with 2-mm mesh in the field. The samples were stored at −20°C until DNA extraction. In laboratory conditions, 0.4 g of soil (fresh weight) was subjected to DNA extraction with FastDNA SPIN Kit for Soil (Q-BioGene).

### DNA amplification and sequencing

Profiling of soil microbial biodiversity was performed by targeting archaea, bacteria, and fungi. For the amplification of the 16S rRNA V4 region of archaea and bacteria (prokaryotes), the set of the forward primer 515f (5′- GTG YCA GCM GCC GCG GTA A -3′) and the reverse primer 806rB (5′- GGA CTA CNV GGG TWT CTA AT -3′) were used as described elsewhere (42). The primers were fused with 3–6-mer Ns for improved Illumina sequencing quality and Illumina sequencing primers. PCR was performed using KOD ONE PCR Master Mix (TOYOBO, Osaka) with the temperature profile of 35 cycles at 98°C for 10 seconds (denaturation), 55°C for 5 seconds (annealing of primers), and 68°C for 30 seconds (extension), and a final extension at 68°C for 2 minutes. The ramp rate through the thermal cycles was set to 1°C/s to prevent the generation of chimeric sequences. In the PCR, we added five artificial DNA sequence variants with different concentrations (i.e., standard DNA gradients; 1.0 × 10^−4^, 5.0 × 10^−5^, 2.0 × 10^−5^, 1.0 × 10^−5^, and 5.0 × 10^−6^ nM; Table S1) to the PCR master mix solution as detailed elsewhere (42). By comparing the number of sequencing reads between the artificial standard DNA and real prokaryotic DNA, the concentration of prokaryotic 16S rRNA genes in template DNA samples was calibrated (42).

In addition to the prokaryotic 16S rRNA region, the internal transcribed spacer 1 (ITS1) region of fungi was amplified using the set of the forward primer ITS1F_KYO1 (5′- CTH GGT CAT TTA GAG GAA STA A -3′) and the reverse primer ITS2_KYO2 (5′ - TTY RCT RCG TTC TTC ATC - 3′) (73). PCR was performed using the Illumina sequencing fusion primer design mentioned above with the temperature profile of 35 cycles at 98°C for 10 seconds, 53°C for 5 seconds, and 68°C for 5 seconds, and a final extension at 68°C for 2 minutes (ramp rate = 1°C/s). Newly designed artificial sequence variants (1.0 × 10^−5^, 7.0 × 10^−6^, 5.0 × 10^−6^, 2.0 × 10^−6^, and 1.0 × 10^−6^ nM; Table S1) were added to the PCR master mix as standard DNA gradients for the calibration of the ITS sequence concentrations in the template DNA samples.

The PCR products of the prokaryotic 16S rRNA and fungal ITS1 regions were, respectively, subjected to the additional PCR step for linking Illumina sequencing adaptors and 8-mer sample identifier indexes with the amplicons. The temperature profile in the PCR was eight cycles at 98°C for 10 seconds, 55°C for 5 seconds, and 68°C for 5 seconds, and a final extension at 68°C for 2 minutes. The PCR products were then pooled for each of the 16S rRNA and fungal ITS1 regions after a purification/equalization process with the AMPureXP Kit (Beckman Coulter, Inc., Brea). Primer dimers, which were shorter than 200 bp, were removed from the pooled library by supplemental purification with AMpureXP: the ratio of AMPureXP reagent to the pooled library was set to 0.8 (vol/vol) in this process. The sequencing libraries of the two regions were processed in an Illumina MiSeq sequencer (10% PhiX spike-in). Because the quality of forward sequences is generally higher than that of reverse sequences in Illumina sequencing, we optimized the MiSeq run setting in order to use only forward sequences. Specifically, the run length was set 271 forward (R1) and 31 reverse (R4) cycles to enhance forward sequencing data: the reverse sequences were used only for discriminating between prokaryotic 16S and fungal ITS1 sequences in the following bioinformatic pipeline.

### Bioinformatics

In total, 23,573,405 sequencing reads were obtained in the Illumina sequencing (16S rRNA, 11,647,166 sequencing reads; ITS, 11,926,239 sequencing reads). The raw sequencing data were converted into FASTQ files using the program bcl2fastq 1.8.4 distributed by Illumina. For each of the 16S rRNA and fungal ITS1 regions, the output FASTQ files were demultiplexed using Claident v0.9.2022.01.26 (74). The sequencing data were deposited to DNA Data Bank of Japan (DDBJ DRA accession no.: DRA015491 and DRA015506). The removal of low-quality sequences and OTU inferences was done using DADA2 (75) v1.17.5 of R v3.6.3 (option settings: maxN = 0, maxEE = 2, truncQ = 11, minLen = 150). The mean number of filtered sequencing reads obtained per sample was 3,949 and 4,075 for the prokaryotic and fungal data sets, respectively. The amplicon sequence variants (ASVs) obtained from the DADA2 pipeline were clustered using the vsearch v2.21.1 program (76) with the 98% and 97% cutoff sequence similarity for prokaryotes and fungi, respectively. Taxonomic annotation of the obtained prokaryotic and fungal OTUs was conducted based on the SILVA 138 SSU (77) and the UNITE all_ 25.07.2023 (78) databases, respectively, with the assignTaxonomy function of DADA2. The OTUs that were not assigned to the domain Archaea/Bacteria and the kingdom Fungi were removed from the 16S rRNA and ITS1 data sets, respectively. Mitochondrial and chloroplast sequences were removed as well in this process. For each target organismal group (prokaryotes and fungi), we then obtained a sample × OTU matrix, in which a cell entry depicted the number of sequencing reads of an OTU in a sample. The samples with less than 1,000 reads were discarded from the matrices. The number of reads was insufficient for comprehensively profiling rare microbial species, which are often targets of soil microbiome studies. However, because data matrices including numerous rare OTUs could not be subjected to the computationally intensive ecological analyses detailed below even if we used supercomputers, we focused on major components of soil prokaryotic and fungal biomes. In other words, our purpose here was to extract major components of agroecosystem soil microbiomes across the Japan Archipelago, thereby finding core microbiome properties associated with disease-suppressive and disease-susceptible agroecosystems. For the sample × OTU matrix, centered log-ratio (CLR) transformation (79–81) was performed using the ALDEx2 v1.35.0 package (82) of R.

In total, prokaryotic and fungal community data were obtained for 2,676 and 2,477 samples, respectively. For fungal OTUs, putative functional groups (e.g., “plant pathogen”) were inferred using the program FungalTraits (83). The estimation of DNA concentrations of the prokaryotic 16S rRNA and fungal ITS regions was performed, respectively, based on the calibration with the standard DNA gradients (artificial DNA variants introduced to the PCR master mix solutions) using the bioinformatic pipeline detailed elsewhere (42).

### Calculation of prokaryote/fungus ratio

Based on the estimated concentrations of prokaryotic 16S rRNA and fungal ITS sequences in template DNA solutions (42, 84), we calculated the ratio of prokaryotic DNA concentrations to fungal DNA concentrations in respective samples (prokaryote/fungus ratio) as follows:


$$log\left(\frac{prokaryotic\;16S\;rRNA\;gene\;concentration\left(DNA\;copies/\mu L\right)}{fungal\;ITS\;gene\;concentration\;\left(DNA\;copies/\mu L\right)}\right).$$

Although potential variation in DNA extraction skills of researchers might affect absolute DNA concentrations in the template DNA solutions, the balance between prokaryotic and fungal DNA in each template DNA sample could be used as a reliable measure. The DNA-metabarcoding-based approach of estimating prokaryote/fungus ratio has a methodological advantage over quantitative-PCR-based approaches. Specifically, the former approach allows us to eliminate effects of nonspecific PCR amplification based on DNA sequencing data, while the latter is affected by “contamination” of nontarget amplicons (e.g., plastid DNA in 16S rRNA sequencing and plant DNA in ITS sequencing).

### Microbiome structure and crop disease prevalence

For each of the prokaryotic and fungal data sets, permutational analysis of variance (PERMANOVA) (85) was performed to examine association between family-level community compositions and variables in the metadata. Two types of PERMANOVA models were constructed based on the Euclidean distance (β-diversity) calculated for the CLR-transformed data sets (1,000 iterations). Specifically, one is constituted by categorical explanatory variables (crop plant, former crop plant, soil taxonomy, research site, and sampling month), while the other included continuous explanatory variables (soil pH, electrical conductivity, C/N ratio, and available phosphorous concentration, prokaryote/fungus ratio, latitude, and longitude).

To reduce the dimensions of the community compositional data, a principal component analysis (PCA) was performed based on the Euclidean distance data mentioned above. For each PCA axis (axes 1–5) in each of the prokaryotic and fungal analyses, Pearson’s correlation with each chemical environmental factor (soil pH, electrical conductivity, C/N ratio, and available phosphorous concentration) was calculated.

We then evaluated how community structure of prokaryotes and fungi was associated with crop disease. For each of the prokaryotic and fungal data sets, a generalized linear mixed model of crop-disease level was constructed by including the PCoA axes 1–5 as fixed effects. Given that disease symptoms could differ greatly among crop plant species, we simplified the classification of disease severity. Specifically, in the GLMM, we set only two categories, i.e., samples showing minimal crop damage (disease level 1) and those showing more severe damage (disease levels 2–5). Sampling month and the identity of crop plant species and experimental/research purposes in the metadata were set as random effects. A logit-link function and binomial errors were assumed in the GLMM after converting the response variable into a binary format (disease level 1 [= 1] vs disease levels 2–5 [= 0]). The analysis was performed with the “glmer” function of the R lme4 package (86).

To confirm the above results based on the Euclidean distance calculated for the CLR-transformed sample × OTU matrices, we performed a supplementary analysis with ordinary approaches based on Bray-Curtis distance for rarefied sample × ASV matrices. Because qualitatively and quantitatively similar statistical results were obtained between the two alternative approaches, the results based on the CLR-transformation approach are shown in the Results section.

### Microbes associated with crop disease/health status

For each microbial OTU constituting the modules, we evaluated the specificity of occurrences in samples differing in crop disease levels based on a randomization analysis. For the calculation, the original sample × OTU matrices of prokaryotes and fungi were, respectively, rarefied to 1,000 reads per sample, being merged into an input data matrix. Within the combined sample × OTU matrix, samples were categorized into the two crop disease levels (disease level 1 vs disease levels 2–5). Mean read counts across samples displaying each of the two disease levels were then calculated for each OTU. Meanwhile, mean read counts for respective disease levels were calculated as well for randomized matrices, in which disease labels of the samples were shuffled (10,000 permutations). For *i*th OTU, standardized specificity to disease level *j* ($s_{ij}$) was obtained as follows:


$$s_{ij}=\;\frac{O_{ij}-Mean\left(R_{ij}\right)}{SD\left(R_{ij}\right)},$$

where $O_{ij}$ and $R_{ij}$ are the mean read counts of *i*th OTU across disease-level *j* samples in the observed and randomized matrices, respectively, and Mean (*R*_*ij*_) and SD (*R*_*ij*_) indicate mean and standard deviation across the randomized matrices. The *P* values obtained based on the randomization analysis were adjusted with the Benjamini-Hochberg method (i.e., false discovery rate). The relationship between the standardized specificity index and FDR is shown in Fig. S4. This randomization approach was also applied to the analyses of each OTU’s specificity to crop plant identity and that to experimental/research purpose identity (Fig. S6 to S7).

The specificity of microbial OTUs to crop disease levels was also performed at the local scale. Specifically, in each of the six sub-data sets representing unique combinations of research sites, crop plant species, and experimental/research purposes, the abovementioned randomization analysis was performed: each sub-data set included 69–198 soil samples (Data S2). For the OTUs showing exceptionally strong specificity to the minimal crop disease level (standardized specificity score ≥ 6.0; FDR < 0.0001), supplemental analyses of generalized linear models (GLMs) were conducted. In each GLM of crop disease/health status (disease level 1 vs disease levels 2–5) with a logit-link function with binomial errors, the relative abundance of a target OTU was included as an explanatory variable.

### Microbe-to-microbe network

To infer potential interactions between microbial OTUs, the algorithm of sparse inverse covariance estimation for ecological association inference (SPIEC-EASI) was applied based on the Meinshausen-Bühlmann method as implemented in the SpiecEasi package (40) of R. In total, 2,305 soil samples from which both prokaryotic and fungal community data were available were subjected to the analysis. Note that CLR-transformation was performed internally with the “spiec.easi” function. The network inference based on co-occurrence patterns allowed us to detect pairs of microbial OTUs that potentially interact with each other in facilitative ways and/or those that might share ecological niches (e.g., preference for edaphic factors). Because estimation of co-occurrence patterns was not feasible for rare nodes, the prokaryotic and fungal OTUs that appeared in more than 10% of the sequenced samples were included in the input matrix of the network analysis. Network modules, within which closely associated OTUs were interlinked with each other, were identified with the algorithm based on edge betweenness (87) using the igraph package (88) of R. For each module in the inferred co-occurrence network, mean standardized specificity to disease level 1 was calculated across constituent OTUs.

To explore potential keystone microbes within the network, we scored respective OTUs on the basis of their topological positions. Among the indices used for evaluating OTUs, betweenness centrality (89), which measures the extent to which a given node (OTU) is located within the shortest paths connecting pairs of other nodes in a network, is commonly used to find hubs mediating flow of effects in a network. The network centrality scores were normalized as implemented in the igraph packages of R. In addition, by focusing on the abovementioned network modules, we ranked OTUs based on their within-module degree and among-module connectivity (90). The former index is obtained as the number of nodes linked with a target node within a target network module, suggesting the topological importance of a node within the module it belongs to. The latter index represents the extent to which a node is linked with other nodes belonging to different network modules. Within-module degree was *z*-standardized (i.e., zero-mean and unit-variance) within each module, while among-module connectivity was defined to vary between 0 and 1. In addition to those indices for evaluating topological roles within a network, eigenvector centrality (91) was calculated for respective nodes.

## Data Availability

The 16S rRNA and ITS sequencing data are available from the DNA Data Bank of Japan (DDBJ accession: DRA015491 and DRA015506). The microbial community data are deposited at our GitHub repository (https://github.com/hiro-toju/Soil_Microbiome_NARO3000). All the R scripts used to analyze the data are available at the GitHub repository (https://github.com/hiro-toju/Soil_Microbiome_NARO3000).

## SUPPLEMENTAL MATERIAL

Data S1 msphere.00803-23-s0001.xlsx Metadata of the soil samples.

Data S2 msphere.00803-23-s0002.xlsx Each OTU's specificity to disease levels.

Data S3 msphere.00803-23-s0003.xlsx Properties of the microbial OTUs within the network.

Supplemental Figures msphere.00803-23-s0004.pdf Figures S1-S9.

Table S1 msphere.00803-23-s0005.pdf Artificial sequences used for the calibration of prokaryote/fungal DNA concentrations.

ASM does not own the copyrights to Supplemental Material that may be linked to, or accessed through, an article. The authors have granted ASM a non-exclusive, world-wide license to publish the Supplemental Material files. Please contact the corresponding author directly for reuse.

## References

[1] Springmann M, Clark M, Mason-D’Croz D, Wiebe K, Bodirsky BL, Lassaletta L, de Vries W, Vermeulen SJ, Herrero M, Carlson KM, Jonell M, Troell M, DeClerck F, Gordon LJ, Zurayk R, Scarborough P, Rayner M, Loken B, Fanzo J, Godfray HCJ, Tilman D, Rockström J, Willett W. 2018. Options for keeping the food system within environmental limits. Nature 562:519–525. doi:10.1038/s41586-018-0594-0 30305731

[2] Tilman D, Balzer C, Hill J, Befort BL. 2011. Global food demand and the sustainable intensification of agriculture. Proc Natl Acad Sci U S A 108:20260–20264. doi:10.1073/pnas.1116437108 22106295 PMC3250154

[3] Toju H, Peay KG, Yamamichi M, Narisawa K, Hiruma K, Naito K, Fukuda S, Ushio M, Nakaoka S, Onoda Y, Yoshida K, Schlaeppi K, Bai Y, Sugiura R, Ichihashi Y, Minamisawa K, Kiers ET. 2018. Core microbiomes for sustainable agroecosystems. Nat Plants 4:247–257. doi:10.1038/s41477-018-0139-4 29725101

[4] Busby PE, Soman C, Wagner MR, Friesen ML, Kremer J, Bennett A, Morsy M, Eisen JA, Leach JE, Dangl JL. 2017. Research priorities for harnessing plant microbiomes in sustainable agriculture. PLoS Biol 15:e2001793. doi:10.1371/journal.pbio.2001793 28350798 PMC5370116

[5] Berendsen RL, Pieterse CMJ, Bakker PAHM. 2012. The rhizosphere microbiome and plant health. Trends Plant Sci 17:478–486. doi:10.1016/j.tplants.2012.04.001 22564542

[6] Bommarco R, Kleijn D, Potts SG. 2013. Ecological intensification: harnessing ecosystem services for food security. Trends Ecol Evol 28:230–238. doi:10.1016/j.tree.2012.10.012 23153724

[7] French E, Kaplan I, Iyer-Pascuzzi A, Nakatsu CH, Enders L. 2021. Emerging strategies for precision microbiome management in diverse agroecosystems. Nat Plants 7:256–267. doi:10.1038/s41477-020-00830-9 33686226

[8] Busby PE, Ridout M, Newcombe G. 2016. Fungal endophytes: modifiers of plant disease. Plant Mol Biol 90:645–655. doi:10.1007/s11103-015-0412-0 26646287

[9] Santoyo G, Moreno-Hagelsieb G, Orozco-Mosqueda M del C, Glick BR. 2016. Plant growth-promoting bacterial endophytes. Microbiol Res 183:92–99. doi:10.1016/j.micres.2015.11.008 26805622

[10] Carey CJ, Dove NC, Beman JM, Hart SC, Aronson EL. 2016. Meta-analysis reveals ammonia-oxidizing bacteria respond more strongly to nitrogen addition than ammonia-oxidizing archaea. Soil Biol Biochem 99:158–166. doi:10.1016/j.soilbio.2016.05.014

[11] Schauss K, Focks A, Leininger S, Kotzerke A, Heuer H, Thiele-Bruhn S, Sharma S, Wilke BM, Matthies M, Smalla K, Munch JC, Amelung W, Kaupenjohann M, Schloter M, Schleper C. 2009. Dynamics and functional relevance of ammonia-oxidizing archaea in two agricultural soils. Environ Microbiol 11:446–456. doi:10.1111/j.1462-2920.2008.01783.x 19196275

[12] Chen M, Arato M, Borghi L, Nouri E, Reinhardt D. 2018. Beneficial services of arbuscular mycorrhizal fungi – from ecology to application. Front Plant Sci 9:1270. doi:10.3389/fpls.2018.01270 30233616 PMC6132195

[13] Baker BJ, De Anda V, Seitz KW, Dombrowski N, Santoro AE, Lloyd KG. 2020. Diversity, ecology and evolution of archaea. Nat Microbiol 5:887–900. doi:10.1038/s41564-020-0715-z 32367054

[14] van der Heijden MGA, Klironomos JN, Ursic M, Moutoglis P, Streitwolf-Engel R, Boller T, Wiemken A, Sanders IR. 1998. Mycorrhizal fungal diversity determines plant biodiversity, ecosystem variability and productivity. Nature 396:69–72. doi:10.1038/23932

[15] Yuan J, Wen T, Zhang H, Zhao M, Penton CR, Thomashow LS, Shen Q. 2020. Predicting disease occurrence with high accuracy based on soil macroecological patterns of Fusarium wilt. ISME J 14:2936–2950. doi:10.1038/s41396-020-0720-5 32681158 PMC7784920

[16] Mendes R, Kruijt M, de Bruijn I, Dekkers E, van der Voort M, Schneider JHM, Piceno YM, DeSantis TZ, Andersen GL, Bakker PAHM, Raaijmakers JM. 2011. Deciphering the rhizosphere microbiome for disease-suppressive bacteria. Science 332:1097–1100. doi:10.1126/science.1203980 21551032

[17] Schlatter D, Kinkel L, Thomashow L, Weller D, Paulitz T. 2017. Disease suppressive soils: new insights from the soil microbiome. Phytopathology 107:1284–1297. doi:10.1094/PHYTO-03-17-0111-RVW 28650266

[18] Wei Z, Gu Y, Friman V-P, Kowalchuk GA, Xu Y, Shen Q, Jousset A. 2019. Initial soil microbiome composition and functioning predetermine future plant health. Sci Adv 5:eaaw0759. doi:10.1126/sciadv.aaw0759 31579818 PMC6760924

[19] Hori Y, Fujita H, Hiruma K, Narisawa K, Toju H. 2021. Synergistic and offset effects of fungal species combinations on plant performance. Front Microbiol 12:713180. doi:10.3389/fmicb.2021.713180 34594312 PMC8478078

[20] Hardoim PR, van Overbeek LS, Elsas JD van. 2008. Properties of bacterial endophytes and their proposed role in plant growth. Trends Microbiol 16:463–471. doi:10.1016/j.tim.2008.07.008 18789693

[21] Kiers ET, Duhamel M, Beesetty Y, Mensah JA, Franken O, Verbruggen E, Fellbaum CR, Kowalchuk GA, Hart MM, Bago A, Palmer TM, West SA, Vandenkoornhuyse P, Jansa J, Bücking H. 2011. Reciprocal rewards stabilize cooperation in the mycorrhizal symbiosis. Science 333:880–882. doi:10.1126/science.1208473 21836016

[22] Jiao S, Yang Y, Xu Y, Zhang J, Lu Y. 2020. Balance between community assembly processes mediates species coexistence in agricultural soil microbiomes across eastern China. ISME J 14:202–216. doi:10.1038/s41396-019-0522-9 31611655 PMC6908645

[23] Ray P, Lakshmanan V, Labbé JL, Craven KD. 2020. Microbe to microbiome: a paradigm shift in the application of microorganisms for sustainable agriculture. Front Microbiol 11:622926. doi:10.3389/fmicb.2020.622926 33408712 PMC7779556

[24] Hartman K, van der Heijden MGA, Wittwer RA, Banerjee S, Walser J-C, Schlaeppi K. 2018. Cropping practices manipulate abundance patterns of root and soil microbiome members paving the way to smart farming. Microbiome 6:14. doi:10.1186/s40168-017-0389-9 29338764 PMC5771023

[25] Shen Z, Xue C, Penton CR, Thomashow LS, Zhang N, Wang B, Ruan Y, Li R, Shen Q. 2019. Suppression of banana panama disease induced by soil microbiome reconstruction through an integrated agricultural strategy. Soil Biol Biochem 128:164–174. doi:10.1016/j.soilbio.2018.10.016

[26] Carrión VJ, Perez-Jaramillo J, Cordovez V, Tracanna V, de Hollander M, Ruiz-Buck D, Mendes LW, van Ijcken WFJ, Gomez-Exposito R, Elsayed SS, Mohanraju P, Arifah A, van der Oost J, Paulson JN, Mendes R, van Wezel GP, Medema MH, Raaijmakers JM. 2019. Pathogen-induced activation of disease-suppressive functions in the endophytic root microbiome. Science 366:606–612. doi:10.1126/science.aaw9285 31672892

[27] Santos LF, Olivares FL. 2021. Plant microbiome structure and benefits for sustainable agriculture. Curr Plant Biol 26:100198. doi:10.1016/j.cpb.2021.100198

[28] Wen T, Xie P, Penton CR, Hale L, Thomashow LS, Yang S, Ding Z, Su Y, Yuan J, Shen Q. 2022. Specific metabolites drive the deterministic assembly of diseased rhizosphere microbiome through weakening microbial degradation of autotoxin. Microbiome 10:177. doi:10.1186/s40168-022-01375-z 36271396 PMC9587672

[29] Tedersoo L, Bahram M, Põlme S, Kõljalg U, Yorou NS, Wijesundera R, Ruiz LV, Vasco-Palacios AM, Thu PQ, Suija A, et al.. 2014. Global diversity and geography of soil fungi. Science 346:1256688. doi:10.1126/science.1256688 25430773

[30] Delgado-Baquerizo M, Oliverio AM, Brewer TE, Benavent-González A, Eldridge DJ, Bardgett RD, Maestre FT, Singh BK, Fierer N. 2018. A global atlas of the dominant bacteria found in soil. Science 359:320–325. doi:10.1126/science.aap9516 29348236

[31] Egidi E, Delgado-Baquerizo M, Plett JM, Wang J, Eldridge DJ, Bardgett RD, Maestre FT, Singh BK. 2019. A few Ascomycota taxa dominate soil fungal communities worldwide. Nat Commun 10:2369. doi:10.1038/s41467-019-10373-z 31147554 PMC6542806

[32] Bahram M, Hildebrand F, Forslund SK, Anderson JL, Soudzilovskaia NA, Bodegom PM, Bengtsson-Palme J, Anslan S, Coelho LP, Harend H, Huerta-Cepas J, Medema MH, Maltz MR, Mundra S, Olsson PA, Pent M, Põlme S, Sunagawa S, Ryberg M, Tedersoo L, Bork P. 2018. Structure and function of the global topsoil microbiome. Nature 560:233–237. doi:10.1038/s41586-018-0386-6 30069051

[33] Faust K, Raes J. 2012. Microbial interactions: from networks to models. Nat Rev Microbiol 10:538–550. doi:10.1038/nrmicro2832 22796884

[34] van der Heijden MGA, Hartmann M. 2016. Networking in the plant microbiome. PLoS Biol 14:e1002378. doi:10.1371/journal.pbio.1002378 26871440 PMC4752285

[35] Saleem M, Hu J, Jousset A. 2019. More than the sum of its parts: microbiome Biodiversity as a driver of plant growth and soil health. Annu Rev Ecol Evol Syst 50:145–168. doi:10.1146/annurev-ecolsys-110617-062605

[36] Durán P, Thiergart T, Garrido-Oter R, Agler M, Kemen E, Schulze-Lefert P, Hacquard S. 2018. Microbial interkingdom interactions in roots promote arabidopsis survival. Cell 175:973–983. doi:10.1016/j.cell.2018.10.020 30388454 PMC6218654

[37] Toju H, Tanaka Y. 2019. Consortia of anti-nematode fungi and bacteria in the rhizosphere of soybean plants attacked by root-knot nematodes. R Soc Open Sci 6:181693. doi:10.1098/rsos.181693 31032023 PMC6458363

[38] Legrand F, Chen W, Cobo-Díaz JF, Picot A, Floch GL. 2019. Co-occurrence analysis reveal that biotic and abiotic factors influence soil fungistasis against Fusarium graminearum. FEMS Microbiol Ecol 95:fiz056. doi:10.1093/femsec/fiz056 30998232

[39] Kerdraon L, Barret M, Laval V, Suffert F. 2019. Differential dynamics of microbial community networks help identify microorganisms interacting with residue-borne pathogens: the case of Zymoseptoria tritici in wheat. Microbiome 7:125. doi:10.1186/s40168-019-0736-0 31470910 PMC6717385

[40] Kurtz ZD, Müller CL, Miraldi ER, Littman DR, Blaser MJ, Bonneau RA. 2015. Sparse and compositionally robust inference of microbial ecological networks. PLoS Comput Biol 11:e1004226. doi:10.1371/journal.pcbi.1004226 25950956 PMC4423992

[41] Toju H, Yamamoto S, Tanabe AS, Hayakawa T, Ishii HS. 2016. Network modules and hubs in plant-root fungal biomes. J R Soc Interface 13:20151097–20151097. doi:10.1098/rsif.2015.1097 26962029 PMC4843674

[42] Fujita H, Ushio M, Suzuki K, Abe MS, Yamamichi M, Iwayama K, Canarini A, Hayashi I, Fukushima K, Fukuda S, Kiers ET, Toju H. 2023. Alternative stable states, nonlinear behavior, and predictability of microbiome dynamics. Microbiome 11:63. doi:10.1186/s40168-023-01474-5 36978146 PMC10052866

[43] Summerell BA. 2019. Resolving Fusarium: current status of the genus. Annu Rev Phytopathol 57:323–339. doi:10.1146/annurev-phyto-082718-100204 31226019

[44] Gordon TR. 2017. Fusarium oxysporum and the Fusarium wilt syndrome. Annu Rev Phytopathol 55:23–39. doi:10.1146/annurev-phyto-080615-095919 28489498

[45] de Lamo FJ, Takken FLW. 2020. Biocontrol by Fusarium oxysporum using endophyte-mediated resistance. Front Plant Sci 11:37. doi:10.3389/fpls.2020.00037 32117376 PMC7015898

[46] Toju H, Yamamichi M, Guimarães PR Jr, Olesen JM, Mougi A, Yoshida T, Thompson JN. 2017. Species-rich networks and eco-evolutionary synthesis at the metacommunity level. Nat Ecol Evol 1:0024. doi:10.1038/s41559-016-0024 28812622

[47] Lebedeva EV, Hatzenpichler R, Pelletier E, Schuster N, Hauzmayer S, Bulaev A, Grigor’eva NV, Galushko A, Schmid M, Palatinszky M, Le Paslier D, Daims H, Wagner M. 2013. Enrichment and genome sequence of the group I.1a ammonia-oxidizing archaeon “Ca. Nitrosotenuis uzonensis” representing a clade globally distributed in thermal habitats. PLoS One 8:e80835. doi:10.1371/journal.pone.0080835 24278328 PMC3835317

[48] Olanrewaju OS, Babalola OO. 2019. Streptomyces: implications and interactions in plant growth promotion. Appl Microbiol Biotechnol 103:1179–1188. doi:10.1007/s00253-018-09577-y 30594952 PMC6394478

[49] Li F, Chen L, Redmile‐Gordon M, Zhang J, Zhang C, Ning Q, Li W. 2018. Mortierella elongata’s roles in organic agriculture and crop growth promotion in a mineral soil. Land Degrad Dev 29:1642–1651. doi:10.1002/ldr.2965

[50] Ozimek E, Hanaka A. 2021. Mortierella species as the plant growth-promoting fungi present in the agricultural soils. Agriculture 11:7. doi:10.3390/agriculture11010007

[51] Poveda J. 2021. Trichoderma as biocontrol agent against pests: new uses for a mycoparasite. Biological Control 159:104634. doi:10.1016/j.biocontrol.2021.104634

[52] Sood M, Kapoor D, Kumar V, Sheteiwy MS, Ramakrishnan M, Landi M, Araniti F, Sharma A. 2020. Trichoderma: the “secrets” of a multitalented biocontrol agent. Plants 9:762. doi:10.3390/plants9060762 32570799 PMC7355703

[53] AbdElfatah HAS, Sallam NMA, Mohamed MS, Bagy HMMK. 2021. Curvularia lunata as new causal pathogen of tomato early blight disease in Egypt. Mol Biol Rep 48:3001–3006. doi:10.1007/s11033-021-06254-8 33687701

[54] Pétriacq P, Stassen JHM, Ton J. 2016. Spore density determines infection strategy by the plant pathogenic fungus Plectosphaerella cucumerina. Plant Physiol 170:2325–2339. doi:10.1104/pp.15.00551 26842622 PMC4825148

[55] Ushio M. 2022. Interaction capacity as a potential driver of community diversity. Proc Soc B 289:20212690. doi:10.1098/rspb.2021.2690 PMC8864351 35193401

[56] Faust K, Lahti L, Gonze D, de Vos WM, Raes J. 2015. Metagenomics meets time series analysis: unraveling microbial community dynamics. Curr Opin Microbiol 25:56–66. doi:10.1016/j.mib.2015.04.004 26005845

[57] Menta C, Remelli S. 2020. Soil health and arthropods: from complex system to worthwhile investigation. Insects 11:54. doi:10.3390/insects11010054 31963103 PMC7022451

[58] Liu T, Chen X, Gong X, Lubbers IM, Jiang Y, Feng W, Li X, Whalen JK, Bonkowski M, Griffiths BS, Hu F, Liu M. 2019. Earthworms coordinate soil biota to improve multiple ecosystem functions. Curr Biol 29:3420–3429. doi:10.1016/j.cub.2019.08.045 31587999

[59] Neher DA. 2010. Ecology of plant and free-living nematodes in natural and agricultural soil. Annu Rev Phytopathol 48:371–394. doi:10.1146/annurev-phyto-073009-114439 20455699

[60] Geisen S, Mitchell EAD, Adl S, Bonkowski M, Dunthorn M, Ekelund F, Fernández LD, Jousset A, Krashevska V, Singer D, Spiegel FW, Walochnik J, Lara E. 2018. Soil protists: a fertile frontier in soil biology research. FEMS Microbiol Rev 42:293–323. doi:10.1093/femsre/fuy006 29447350

[61] Zhao ZB, He JZ, Geisen S, Han LL, Wang JT, Shen JP, Wei WX, Fang YT, Li PP, Zhang LM. 2019. Protist communities are more sensitive to nitrogen fertilization than other microorganisms in diverse agricultural soils. Microbiome 7:33. doi:10.1186/s40168-019-0647-0 30813951 PMC6393985

[62] Kageyama T, Toju H. 2022. Effects of source sample amount on biodiversity surveys of bacteria, fungi, and nematodes in soil ecosystems. Front Ecol Evol 10:959945. doi:10.3389/fevo.2022.959945

[63] Quince C, Walker AW, Simpson JT, Loman NJ, Segata N. 2017. Shotgun metagenomics, from sampling to analysis. Nat Biotechnol 35:833–844. doi:10.1038/nbt.3935 28898207

[64] Neal AL, Barrat HA, Bacq-Lebreuil A, Qin Y, Zhang X, Takahashi T, Rubio V, Hughes D, Clark IM, Cárdenas LM, Gardiner LJ, Krishna R, Glendining ML, Ritz K, Mooney SJ, Crawford JW. 2023. Arable soil nitrogen dynamics reflect organic inputs via the extended composite phenotype. Nat Food 4:51–60. doi:10.1038/s43016-022-00671-z 37118575

[65] Wu X, Rensing C, Han D, Xiao K-Q, Dai Y, Tang Z, Liesack W, Peng J, Cui Z, Zhang F. 2022. Genome-resolved metagenomics reveals distinct phosphorus acquisition strategies between soil microbiomes. mSystems 7. doi:10.1128/msystems.01107-21 PMC8751388 35014868

[66] Zelezniak A, Andrejev S, Ponomarova O, Mende DR, Bork P, Patil KR. 2015. Metabolic dependencies drive species co-occurrence in diverse microbial communities. Proc Natl Acad Sci U S A 112:6449–6454. doi:10.1073/pnas.1421834112 25941371 PMC4443341

[67] Fujita H, Ushio M, Suzuki K, Abe MS, Yamamichi M, Okazaki Y, Canarini A, Hayashi I, Fukushima K, Fukuda S, Kiers ET, Toju H. 2023. Facilitative interaction networks in experimental microbial community dynamics. Front Microbiol 14:1153952. doi:10.3389/fmicb.2023.1153952 37113242 PMC10126487

[68] Luo G, Li L, Friman VP, Guo J, Guo S, Shen Q, Ling N. 2018. Organic amendments increase crop yields by improving microbe-mediated soil functioning of agroecosystems: a meta-analysis. Soil Biol Biochem 124:105–115. doi:10.1016/j.soilbio.2018.06.002

[69] Kassam A, Friedrich T, Derpsch R. 2019. Global spread of conservation agriculture. Int J Environ Sci 76:29–51. doi:10.1080/00207233.2018.1494927

[70] Schreefel L, Schulte RPO, de Boer IJM, Schrijver AP, van Zanten HHE. 2020. Regenerative agriculture – the soil is the base. Global Food Sec 26:100404. doi:10.1016/j.gfs.2020.100404

[71] Li Y, Zhang Q, Cai Y, Yang Q, Chang SX. 2020. Minimum tillage and residue retention increase soil microbial population size and diversity: implications for conservation tillage. Sci Total Environ 716:137164. doi:10.1016/j.scitotenv.2020.137164 32059331

[72] Chiang KS, Liu HI, Bock CH. 2017. A discussion on disease severity index values. part I: warning on inherent errors and suggestions to maximise accuracy. Ann Appl Biol 171:139–154. doi:10.1111/aab.12362

[73] Toju H, Tanabe AS, Yamamoto S, Sato H. 2012. High-coverage ITS primers for the DNA-based identification of ascomycetes and basidiomycetes in environmental samples. PLoS One 7:e40863. doi:10.1371/journal.pone.0040863 22808280 PMC3395698

[74] Tanabe AS, Toju H. 2013. Two new computational methods for universal DNA barcoding: a benchmark using barcode sequences of bacteria, archaea, animals, fungi, and land plants. PLoS One 8:e76910. doi:10.1371/journal.pone.0076910 24204702 PMC3799923

[75] Callahan BJ, McMurdie PJ, Rosen MJ, Han AW, Johnson AJA, Holmes SP. 2016. DADA2: high-resolution sample inference from illumina amplicon data. Nat Methods 13:581–583. doi:10.1038/nmeth.3869 27214047 PMC4927377

[76] Rognes T, Flouri T, Nichols B, Quince C, Mahé F. 2016. VSEARCH: a versatile open source tool for metagenomics. PeerJ 4:e2584. doi:10.7717/peerj.2584 27781170 PMC5075697

[77] Quast C, Pruesse E, Yilmaz P, Gerken J, Schweer T, Yarza P, Peplies J, Glöckner FO. 2013. The SILVA ribosomal RNA gene database project: improved data processing and web-based tools. Nucleic Acids Res 41:D590–D596. doi:10.1093/nar/gks1219 23193283 PMC3531112

[78] Nilsson RH, Larsson KH, Taylor AFS, Bengtsson-Palme J, Jeppesen TS, Schigel D, Kennedy P, Picard K, Glöckner FO, Tedersoo L, Saar I, Kõljalg U, Abarenkov K. 2019. The UNITE database for molecular identification of fungi: handling dark taxa and parallel taxonomic classifications. Nucleic Acids Res 47:D259–D264. doi:10.1093/nar/gky1022 30371820 PMC6324048

[79] Quinn TP, Erb I, Gloor G, Notredame C, Richardson MF, Crowley TM. 2019. A field guide for the compositional analysis of any-omics data. Gigascience 8:1–14. doi:10.1093/gigascience/giz107 PMC6755255 31544212

[80] Gloor GB, Macklaim JM, Pawlowsky-Glahn V, Egozcue JJ. 2017. Microbiome datasets are compositional: and this is not optional. Front Microbiol 8:2224. doi:10.3389/fmicb.2017.02224 29187837 PMC5695134

[81] Aitchison J, Barceló-Vidal C, Martín-Fernández JA, Pawlowsky-Glahn V. 2000. Logratio analysis and compositional distance. Math Geol 32:271–275. doi:10.1023/A:1007529726302

[82] Fernandes AD, Reid JNS, Macklaim JM, McMurrough TA, Edgell DR, Gloor GB. 2014. Unifying the analysis of high-throughput sequencing datasets: characterizing RNA-seq, 16S rRNA gene sequencing and selective growth experiments by compositional data analysis. Microbiome 2:15. doi:10.1186/2049-2618-2-15 24910773 PMC4030730

[83] Põlme S, Abarenkov K, Henrik Nilsson R, Lindahl BD, Clemmensen KE, Kauserud H, Nguyen N, Kjøller R, Bates ST, Baldrian P, et al.. 2020. FungalTraits: a user-friendly traits database of fungi and fungus-like stramenopiles. Fungal Divers 105:1–16. doi:10.1007/s13225-020-00466-2

[84] Yajima D, Fujita H, Hayashi I, Shima G, Suzuki K, Toju H. 2023. Core species and interactions prominent in fish-associated microbiome dynamics. Microbiome 11:53. doi:10.1186/s40168-023-01498-x 36941627 PMC10026521

[85] Anderson MJ. 2001. A new method for non-parametric multivariate analysis of variance. Austral Ecol 26:32–46. doi:10.1046/j.1442-9993.2001.01070.x

[86] Bates D, Mächler M, Bolker BM, Walker SC. 2015. Fitting linear mixed-effects models using lme4. J Stat Softw 67:1–48. doi:10.18637/jss.v067.i01

[87] Newman MEJ, Girvan M. 2004. Finding and evaluating community structure in networks. Phys Rev E 69:026113. doi:10.1103/PhysRevE.69.026113 14995526

[88] Csardi G, Nepusz T. 2006. The igraph software package for complex network research. InterJ Complex Syst 1695:1–9.

[89] Freeman LC. 1977. A set of measures of centrality based on betweenness. Sociometry 40:35. doi:10.2307/3033543

[90] Olesen JM, Bascompte J, Dupont YL, Jordano P. 2007. The modularity of pollination networks. Proc Natl Acad Sci U S A 104:19891–19896. doi:10.1073/pnas.0706375104 18056808 PMC2148393

[91] Bonacich P. 1972. Factoring and weighting approaches to status scores and clique identification. J Mathematical Soc 2:113–120. doi:10.1080/0022250X.1972.9989806

