# Supplementary material for: Soil prokaryotic and fungal biome structures associated with crop disease status across the Japan Archipelago
Source: mSphere. 2024 Apr 3;9(4):e00803-23. doi: 10.1128/msphere.00803-23 (PMC11036807; doi:10.1128/msphere.00803-23)
Supplement: Table S1 — Artificial sequences used for the calibration of prokaryote/fungal DNA concentrations. [file msphere.00803-23-s0005.pdf]

Table S1 | Artificial sequences used for the calibration of prokaryote/fungal DNA concentrations. The artificial sequences with defined concentrations were mixed with the PCR master mix solutions as standard DNA gradients. The artificial sequences consisted of primer annealing regions, conservative sequence regions adjacent to the primer regions, and random nucleotide sequences at the highly-variable region at the intermediate positions. The standard (artificial) sequences were distinguished in the bioinformatic pipeline and they were used for the estimation (calibration) of prokaryote 16S rRNA or fungal ITS DNA concentrations in template DNA samples as detailed elsewhere[1–3].

Standard DNA variants used in the prokaryote analysis[2].
>STD_pro1
GTGCCAGCAGCCGCGGTAAGACGGAGGGGGCTAGCGTTGTTCGGAATTACTGGGCG
TAAAGAGAAGGTAGGCGGAAGCTGAAGTCATGTGTGAAAACGCCTGGCTTAACTTA
GCTCAGGGTCGCTAAACTGGTTGGCTTGAGTGTGAACGAGGTCCTCGGAATTTTCTG
TGTAGCGGTGAAATGCGTAGATATTAAGGCGAACACCTGCGGCGAAGGCACGGAGC
TGGGGCAGGGCTGACGCTGAGGCGTTAAAGCGTGGGGAGCAAACAGGATTAGATAC
CCTGGTAGTCC

>STD_pro2
GTGCCAGCAGCCGCGGTAAGACGGAGGGGGCTAGCGTTGTTCGGAATTACTGGGCG
TAAAGAGTATGTAGGCGGTAAAGGAAGTTACGAGTGAAATTACAGGGCTTAACCGA
TAAGTCGTGGCCAAAACTGGGAGCCTTGAGTAATCGAGAGGTGGGCGGAATTGGGT
GTGTAGCGGTGAAATGCGTAGATATTCAAAGGAACACCGATCGCGAAGGCGGCCTC
CTGGTTAGGTCCTGACGCTGAGGAACGAAAGCGTGGGGAGCAAACAGGATTAGATA
CCCTGGTAGTCC

>STD_pro3
GTGCCAGCAGCCGCGGTAAGACGGAGGGGGCTAGCGTTGTTCGGAATTACTGGGCG
TAAAGAGTTAGTAGGCGGGCAATTAAGTTATAGGTGAAAAGTAATGGCTTAACTTCG
CGAACTCCGGACAAACTGAGGTGCTTGAGCGTTAAAGAGGCTCCCGGAATTGAAGG
TGTAGCGGTGAAATGCGTAGATATGTTTCGGAACACCTAATGCGAAGGCTCAAGTCT
GGGTAACTGGTGACGCTGAGGTCACAAAGCGTGGGGAGCAAACAGGATTAGATACC
CTGGTAGTCC

>STD_pro4
GTGCCAGCAGCCGCGGTAAGACGGAGGGGGCTAGCGTTGTTCGGAATTACTGGGCG
TAAAGAGAGTGTAGGCGGCCACGTAAGTTCCCGGTGAAATCGAGCGGCTTAACGTG
CTCCTCGCCCGAGAAACTGAAGCCCTTGAGCTCAGCCGAGGAACCCGGAATTACTTG
TGTAGCGGTGAAATGCGTAGATATGTGTATGAACACCTCCAGCGAAGGCCGCACACT
GGCACTCCACTGACGCTGAGGTTTAAAAGCGTGGGGAGCAAACAGGATTAGATACC
CTGGTAGTCC

>STD_pro5

GTGCCAGCAGCCGCGGTAAGACGGAGGGGGCTAGCGTTGTTCGGAATTACTGGGCG
TAAAGAGCCGGTAGGCGGCTCCCGAAGTCGGTAGTGAAATTCTGAGGCTTAACTAA
AACCACGACATTGAAACTGGGTGTCTTGAGTTGATACGAGGCCAGTGGAATTGTGCG
TGTAGCGGTGAAATGCGTAGATATAATAAGGAACACCTCCCGCGAAGGCTTTGGCCT
GGCATGCCAGTGACGCTGAGGTGTTAAAGCGTGGGGAGCAAACAGGATTAGATACC
CTGGTAGTCC

Standard DNA variants used in the fungal analysis (developed in this study)
>STD_fng1
CTTGGTCATTTAGAGGAAGTAAAAGTCGTAACAAGGCTTCCGTAGGTGAACCTGCGC
AAGGATCATTAGGTATCACTCAGGAAGCAGACACAGAAAGACACGGTCTAGCAGAT
CGTTTATCGGCTAGGTCAAATAGAGTGCTTTGATATCAGCATGTCTAGCTTTAGAATT
CAGTTTAGTGCGCTGATCTGAGTCGAGATAAAATCACCAGTACCCAAAACCAGGCGG
GCTCGCCACGTACATCCAACAACGGATCTCTTGGTTCCGGCATCGATGAAGAACGCA
GCGAA

>STD_fng2
CTTGGTCATTTAGAGGAAGTAAAAGTCGTAACAAGGATCCCGTAGGTGAACCTGCGT
AAGGATCATTATCCTGCCCAGTAGCGGATGATAATGGTTGTTGCCAGCCGGTGTGGA
AGGTAACAGCACCGGTGCGAGCCTAATGTGCCGTCTCCACCAACACAAGGCTATCCG
GTCGTATAATAGGATTCCGCAATGGGGTTAGCAAATGGCAGCCTAAACGATATCGGG
GACTTGCGATGACTTGCAACAACGGATCTCTAGGTTCCGGCATCGATGAAGAACGCA
GCGAA

>STD_fng3
CTTGGTCATTTAGAGGAAGTAAAAGTCGTAACAAGGTATCCGTAGGTGAACCTGCGC
AAGGATCATTAGAGGCGTTACCCCAATCGTTCAGCGTGGGATTTGCTACAACTTCTG
AGTGCTACATGTACGAGACCATGTTATGTATGCACAAGGCCGACAATAGGACGTAGC
CTTCGAGTTAGTACGTAGCGTGGTCGCATAAGCACAGTAGATCCTCCCCGCGCATCC
TATTTATTAAGACGTTCAACAACGGATCTCTTGGTTCCGGCATCGATGAAGAACGCA
GCGAA

>STD_fng4
CTTGGTCATTTAGAGGAAGTAAAAGTCGTAACAAGGCTACCGTAGGTGAACCTGCGT
AAGGATCATTATTAATTCTATAGCAATACGATCATATGCGGATGGGCAGTGGCCGGT
AGTCACACGTCTACCGCGGTGCTCAATGACCGGGACTAAAGAGGCGAAGATTATGG
TGTGTGACCCGTTATGCTCGAGTTCGGTCAGAGCGTCATTGCGAGTAGTCGATTGCTT
TCTCAATCTCCACCTACAACAACGGATCTCTGGGTTCCGGCATCGATGAAGAACGCA
GCGAA

>STD_fng5
CTTGGTCATTTAGAGGAAGTAAAAGTCGTAACAAGGTTGCCGTAGGTGAACCTGCGC
AAGGATCATTAGTACAGGTTCGCCTGTCGCCAAGATGCCTTACCTAGATGCAATGAC
GGACGTATTCCTCTGGCCTCAACGGTTCCTGCTTTCGCTGGGATCCAAGATTGGCAGC
TGAAACCGCCTTTCCAAAGTGAGTCCTTCGTCTGTGACTAACTGTGCCAAATCGTCTT

GCAAACTCCACATTCAACAACGGATCTCTCGGTTCCGGCATCGATGAAGAACGCAGC
GAA

References

1. Ushio, M. *et al.* Quantitative monitoring of multispecies fish environmental DNA using high-throughput sequencing. *Metabarcoding Metagenom* **2**, 1–15 (2018).
2. Ushio, M. Interaction capacity as a potential driver of community diversity. *Proceedings of the Royal Society B: Biological Sciences* **289**, 20212690 (2022).
3. Fujita, H. *et al.* Alternative stable states, nonlinear behavior, and predictability of microbiome dynamics. *Microbiome* **11**, 63 (2023).
